# Pattern Dynamics in Adaxial-Abaxial Specific Gene Expression Are Modulated by a Plastid Retrograde Signal during *Arabidopsis thaliana* Leaf Development

**DOI:** 10.1371/journal.pgen.1003655

**Published:** 2013-07-25

**Authors:** Toshiaki Tameshige, Hironori Fujita, Keiro Watanabe, Koichi Toyokura, Maki Kondo, Kiyoshi Tatematsu, Noritaka Matsumoto, Ryuji Tsugeki, Masayoshi Kawaguchi, Mikio Nishimura, Kiyotaka Okada

**Affiliations:** 1 Department of Botany, Kyoto University, Kyoto, Japan; 2 Laboratory of Plant Organ Development, National Institute for Basic Biology, Okazaki, Aichi, Japan; 3 Division of Symbiotic Systems, National Institute for Basic Biology, Okazaki, Aichi, Japan; 4 Division of Cell Mechanisms, National Institute for Basic Biology, Okazaki, Aichi, Japan; 5 School of Life Science, Graduate University for Advanced Studies (Sokendai), Okazaki, Aichi, Japan; University of California Riverside, United States of America

## Abstract

The maintenance and reformation of gene expression domains are the basis for the morphogenic processes of multicellular systems. In a leaf primordium of *Arabidopsis thaliana*, the expression of *FILAMENTOUS FLOWER* (*FIL*) and the activity of the microRNA miR165/166 are specific to the abaxial side. This miR165/166 activity restricts the target gene expression to the adaxial side. The adaxial and abaxial specific gene expressions are crucial for the wide expansion of leaf lamina. The *FIL*-expression and the miR165/166-free domains are almost mutually exclusive, and they have been considered to be maintained during leaf development. However, we found here that the position of the boundary between the two domains gradually shifts from the adaxial side to the abaxial side. The cell lineage analysis revealed that this boundary shifting was associated with a sequential gene expression switch from the *FIL-*expressing (miR165/166 active) to the miR165/166-free (non-*FIL*-expressing) states. Our genetic analyses using the *enlarged fil expression domain2* (*enf2*) mutant and chemical treatment experiments revealed that impairment in the plastid (chloroplast) gene expression machinery retards this boundary shifting and inhibits the lamina expansion. Furthermore, these developmental effects caused by the abnormal plastids were not observed in the *genomes uncoupled1* (*gun1*) mutant background. This study characterizes the dynamic nature of the adaxial-abaxial specification process in leaf primordia and reveals that the dynamic process is affected by the *GUN1*-dependent retrograde signal in response to the failure of plastid gene expression. These findings advance our understanding on the molecular mechanism linking the plastid function to the leaf morphogenic processes.

## Author Summary

The efficient photosynthesis in plant leaves depends on the wide planar morphology of lamina with a lot of chloroplasts (plastids). Development of the planar lamina requires the specific expression of several key genes in the foreside (adaxial side) and backside (abaxial side) of the tiny leaf primordium. Such abaxial-specific genes include *FIL* and miR165/166. In this study, we characterized that the expression of *FIL* and the activity of miR165/166 are induced in all cells of initiating leaf primordia, and then repressed sequentially from the adaxial cells, thus gradually restricted to the abaxial cells. Furthermore, we found that this restriction of *FIL* expression and miR165/166 activity is retarded and lamina becomes narrow when the plastid function is inhibited in leaf primordia. Interestingly, such plastid effects on leaf development were not observed when the communication between plastid and nucleus was inhibited by *gun1* mutation. Our study suggests that plastids modulate the gene expression dynamics in leaf primordia leading to narrow lamina formation when the plastid function is severely inhibited. Such developmental regulation by plastid presumably contributes to preventing the wasteful expansion of lamina with low photosynthetic activity.

## Introduction

The expansion of a flat organ from an undifferentiated organ primordium provides an excellent model for studying the dynamics of formation and maintenance of gene expression domains. In the case of wing development in *Drosophila*, the wing primordium (wing disc) is subdivided into dorsal and ventral compartments. Each compartment specifically expresses key genes determining the future pattern of tissue growth and cell differentiation. Cells in each compartment are related by lineage and they are prevented from crossing the dorso-ventral boundary [1]–[4].

In the case of leaf development in the model plant *Arabidopsis thaliana*, previous studies have revealed that several genes are expressed in an adaxial- or abaxial- specific manner. Their specific expression patterns are required for lamina expansion with adaxial-abaxial asymmetric cell differentiation (see [5]–[8] for review). It has been considered that their expression patterns are established during leaf initiation, that is, stages P0 to P1, and maintained during later stages [5]–[7]. However, previous studies have not focused on whether the gene expression states are maintained in each cell lineage as in the case of the fly wing. The adaxial-specific genes are three *Class III Homeodomain-Leucine Zipper* genes, *PHABULOSA* (*PHB*), *PHAVOLUTA* and *REVOLUTA* (*REV*) (*PHB*-like genes hereafter) [9]–[11], and a LOB-domain family gene, *ASYMMETRIC LEAVES2* [12]. The abaxial-specific genes are four *YABBY* family genes, including *FILAMENTOUS FLOWER* (*FIL*) [13]–[16], three *KANADI* genes (*KANs*) [17], [18] and two *AUXIN RESPONSE FACTOR* genes (*ARFs*) [19], [20]. In addition to such transcription factor genes, small regulatory RNAs are also distributed in an adaxial- or abaxial-specific manner. The microRNA miR165/166 represses the expression of *PHB*-like genes through mRNA cleavage in the abaxial region [21]. The trans-acting small interfering RNAs targeting the two *ARFs* (tasiR-ARFs) also repress their targets through mRNA cleavage in the adaxial region [20], [22]. Especially, the intercellular mobility of these small RNAs has been recently emphasized as the key feature to formation of spatial gene expression patterns [20], [23], [24].

The expression patterns of these transcription factors and small RNAs are considered to be the results from complex regulatory networks among themselves though many parts of the networks are yet to be elucidated [25]. Nonetheless, it has been discussed that the adaxial- and abaxial-specific expression domains of such genes are separated and maintained owing to the mutual repression between these adaxial- and abaxial-specific genes via direct transcriptional repression, mRNA degradation and other negative regulations [5]–[8], [11]. It is highly likely that intracellular mutual repression between two (groups of) genes allows each cell to express only one (group of) gene(s) and maintain the gene expression state. On the other hand, it is not necessarily likely that the intercellular mutual repression between the genes contributes to the maintenance of the gene expression domains. While such intercellular effects prevent a cell from misexpressing the other genes within one gene expression domain, both the two domains might not be maintained for a long time because such intercellular effects might change the gene expression state of the cells on the domain boundary. This speculation is consistent with many studies showing that when two mobile factors decrease each other's quantity, the boundary between their distribution domains shifts in theory and real observations [26]–[28].

In this study, we first performed computer simulations of a simple mathematical model assuming mutual repression between two factors representing the adaxial- and abaxial-specific genes. These simulations showed that the boundary position between their expression domains is not necessarily maintained, but might shift toward one end when the repression is mediated by mobile factors.

It has been described previously that the *FIL*-expression domain is separated from the expression domain of *PHB*-like genes, or the miR165/166-free domain, with no or a little overlap in primordia of leaves [29], flowers [30], cotyledons [31] and sepals [25]. Orthologous genes of *FIL* and *PHB* also show similar complementary expression patterns in *Antirrhinum* [32] and *Cabomba* [33]. Though the previous studies have not clearly shown whether or not the boundary position between these domains changes during leaf development, it has been reported that *FIL*-expression domains differ at different developmental stages of leaves [13], [14], [34]. While the *Arabidopsis* leaves basically consist of six cell layers: the adaxial epidermis, four layers of mesophyll and the abaxial epidermis, *FIL* is initially expressed in the whole leaf at approximately the P0 stage, and then restricted to the abaxial four cell layers at later stages [13], [14]. This expression domain is further restricted to the three abaxial cell layers at approximately the P6 (sixth youngest leaf) stage [34]. The similar gradual restriction of the *FIL* expression to the abaxial cells has been reported in tomatoes [35].

Here we characterized in detail how *FIL*-expression and miR165/166-free (presumptively *PHB-*like genes expression) domains change during leaf development by careful observations of the gene expression markers and by lineage analysis of *FIL*-expressing cells. The results showed that all leaf founder cells express *FIL* and have miR165/166 activity, but the *FIL*-expressing cells become miR165/166-free cells sequentially from the adaxial to the abaxial side after leaf initiation. In other words, the boundary between *FIL*-expression and miR165/166-free domains shifts from the adaxial to the abaxial side similarly to the above computer simulation.

Our genetic analyses demonstrated that excessively fast shifting of the boundary is associated with narrow lamina formation and excess adaxialization in cell differentiation, whereas excessively slow shifting of the boundary is associated with narrow lamina formation and excess abaxialization. Furthermore, detailed analyses of the mutant *enlarged fil expression domain2* (*enf2*) revealed that the boundary shifting is retarded by the *GENOMES UNCOUPLED1-* (*GUN1-*) dependent mechanism when plastid (chloroplast) gene expression machinery is compromised by inhibitor treatments and genetic mutations. *GUN1* is an indispensable factor for the plastid-nucleus communication system known as retrograde signaling (see [36]–[40] for review). Therefore, our results strongly suggest that the *GUN1*-dependent plastid retrograde signal regulates leaf morphogenesis by affecting the dynamic change in the *FIL*-expression and miR165/166-free domains in leaf primordia.

From physiological point of view, the main advantage of lamina expansion is the efficiency in light reception for photosynthesis, thus depends on the functional integrity of plastids. On the other hand, from developmental viewpoint, the lamina expansion depends on the adaxial- and abaxial-specific genes in leaf primordia. We discuss how the link between adaxial-abaxial gene expression and plastid condition contributes to plant growth and development.

## Results

### A Boundary between Two Gene Expression Domains Easily Shifts when Their Products Are Mobile and Repress Each Other's Expression

To know the theoretical stability of neighboring two gene expression domains when the two (groups of) genes repress each other's expression, we examined the domain stability by numerical analysis. Here we suppose a situation where

**Figure 1:**
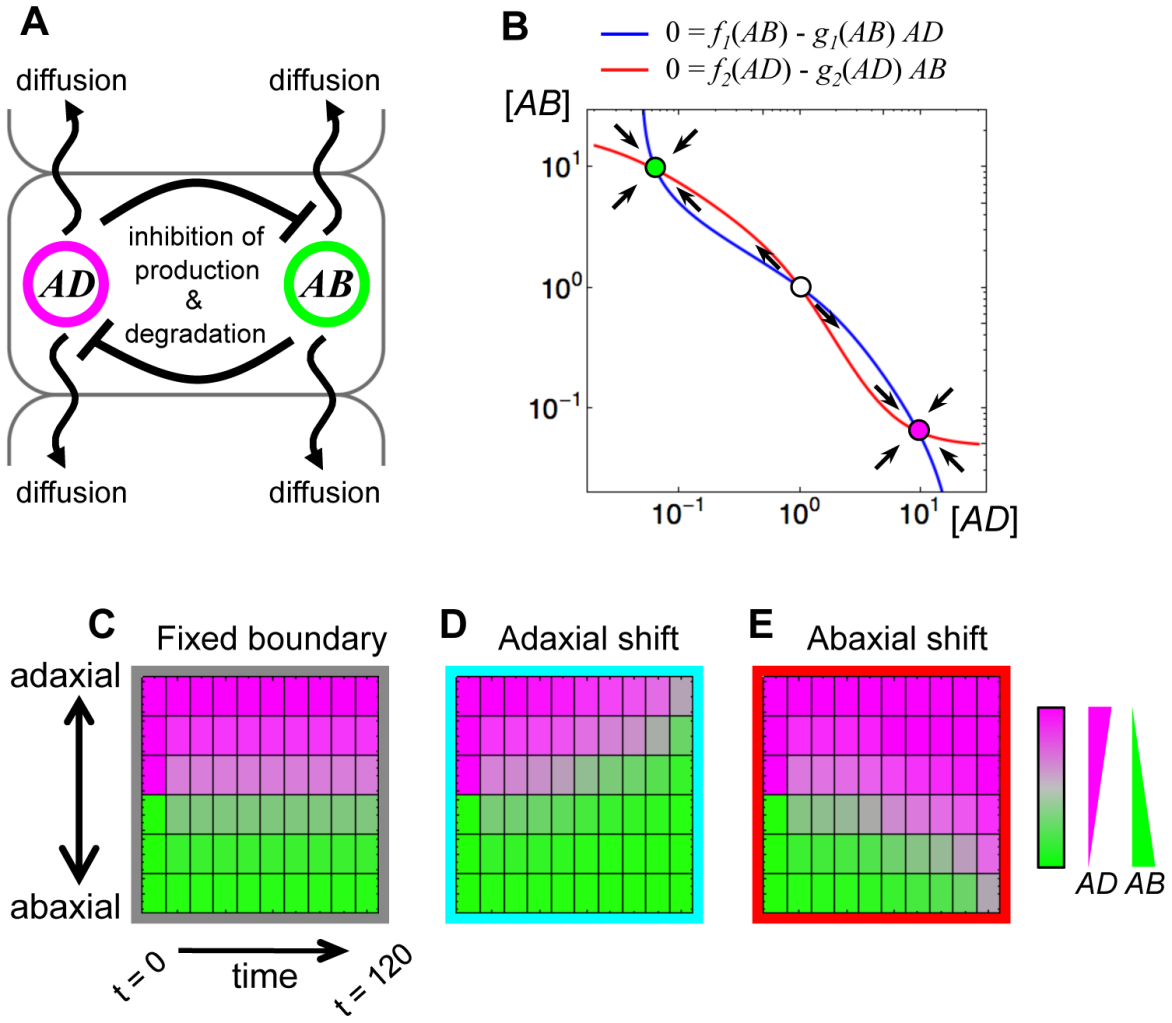
A simple mutual repression of genes mediated by mobile factors easily shifts the boundary between gene expression domains. (A) Schematic illustration of the model of mutual repression and mobility. (B) The phase plane of the mutual repression system. Blue and red lines indicate nullclines of equations (1) and (2), respectively, without the diffusion terms. The filled circles with magenta and green indicate the stable steady states, named the *AD*-expressing state and the *AB*-expressing state, respectively. The open circle is an unstable steady state. (C–E) Simulation results of the mathematical model using three parameter sets: the symmetric parameter set between *AD* and *AB* (C); the asymmetric parameter sets (D, E). The parameter values are set to be *p_1_* = *p_2_* = 0.1, *r_1_* = *r_2_* = 2.0, *d_1_* = *d_2_* = 0.1, *c_1_* = *c_2_* = 2.0 and *D_AD_* = *D_AB_* = 0.1 (C), except for *r_2_* = 1.8 (D), *r_1_* = 1.8 (E).

1. Two gene products: *AD* and *AB*, are produced accordingly to the production functions *f_1_* and *f_2_*, respectively, and degraded accordingly to the degradation functions *g_1_* and *g_2_*, respectively, within each cell.
2. *AD* represses the production and promotes the degradation of *AB*, on the other hand, *AB* represses the production and promotes the degradation of *AD*. Therefore, *f_1_* and *f_2_* are decreasing functions, and *g_1_* and *g_2_* are increasing functions (Figure 1A, S1A, S1B).
3. When only a cell is considered, the cell can predominantly express either of *AD* or *AB* in a stable gene expression state due to the mutual repression between *AD* and *AB*. However, both expression states are stable. (Figure 1B)
4. Cells are aligned in one-dimensional space representing the adaxial-abaxial axis.
5. *AD* and *AB* are uniformly distributed within each cell and moves between neighboring cells in a gradient-dependent manner like the simple diffusion. (Figure 1A)

Thus, the dynamics of *AD* and *AB* in cell “*i*” are formulated as 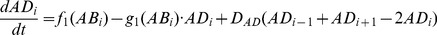
(1)

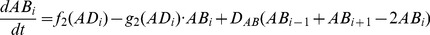
(2)where *D_AD_* and *D_AB_* are constant diffusion coefficients of *AD* and *AB*, respectively. For simplicity, the functions *f* and *g* are described as basal independent constants (the first terms) plus the Hill equations (the second terms).
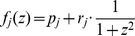
(3)

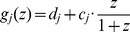
(4)(*j* = 1, 2)

where *p_j_* is the basal production rate, *r_j_* is the inhibitory effect of the production rate (e.g., the effect of transcriptional repression), *d_j_* is the basal degradation rate and *c_j_* is the promotion strength of the degradation rate (e.g., the effect of mRNA cleavage) (Figure S1A, S1B). Such a mutual repression dynamics has the *AD*-expressing and the *AB*-expressing states as stable steady states with a certain range of parameter values (e.g., *p_1_* = *p_2_* = 0.1, *r_1_* = *r_2_* = 2.0, *d_1_* = *d_2_* = 0.1 and *c_1_* = *c_2_* = 2.0, Figure 1B) when only a single cell is considered (Figure 1B).

After such a bistable mathematical model was developed, we lay *AD*-expressing cells and *AB*-expressing cells side by side and simulate the time evolution of *AD* and *AB* expression. Here we consider only three *AD*-expressing cells and three *AB*-expressing cells as the initial states. The results are shown in Figure 1C–E. In the cases where parameters are symmetric between *AD* and *AB* (i.e., *p_1_* = *p_2_*, *r_1_* = *r_2_*, *d_1_* = *d_2_*, *c_1_* = *c_2_* and *D_AD_* = *D_AB_*), the expression domains is maintained, in other wards, the initial domain boundary is fixed (Figure 1C). On the other hand, when a parameter is changed from such symmetric parameter set to asymmetric one, one gene expression domain expands and the other shrinks, namely, the domain boundary shifts toward the either end (e.g., *p_1_* = *p_2_*, *r_1_*>*r_2_*, *d_1_* = *d_2_*, *c_1_* = *c_2_* and *D_AD_* = *D_AB_* for Figure 1D, *p_1_* = *p_2_*, *r_1_*<*r_2_*, *d_1_* = *d_2_*, *c_1_* = *c_2_* and *D_AD_* = *D_AB_* for Figure 1E) (see also Figure S1C–F).

These simulations give examples of the known mathematical rule that negatively interacting mobile factors easily cause boundary shifting between their distribution domains [26], and suggest that this rule can be applied to molecular-biological systems including the regulatory network among the adaxial- and abaxial-specific genes in leaf primordia.

### The Boundary between *FIL*-expression and mir165/166-free Domains Shifts

The almost exclusive relationship between the abaxial *FIL*-expression domain and the adaxial miR165/166-free domain has been characterized well [25], [29]–[31]. However, whether or not these domains are simply maintained during leaf development had not yet been explicitly demonstrated. If the domain separation is due to mutual repression between miR165/166 and *PHB*-like genes, *FIL* and *PHB*-like genes and between upstream regulators for these genes, the domain boundary might not be necessarily maintained.

To characterize in detail the spatio-temporal patterns of the *FIL*-expression and miR165/166-free domains during leaf development, we observed these domains at a series of leaf developmental stages. The *FIL-*expression and miR165/166-free domains were visualized by two fluorescent markers, *FILpro:GFP* (green fluorescent protein driven by the *FIL* promoter) and *35Spro:miYFP-W* (miR165/166-sensor yellow fluorescent protein driven by the Cauliflower Mosaic Virus 35S promoter), respectively, as previously described [29].

Before leaf initiation (around P0 stages), *FIL* was likely expressed in all the leaf founder cells (Figure S2C, S2D), as previously reported [13], [14], and miR165/166 activity was higher in these cells than in the surrounding cells (Figure S2C, S2D). However, it was difficult to determine whether the *FIL*-expressing and miR165/166-active cells are all leaf founder cells because of the absence of a marker to distinguish those cells from the remaining meristem cells in which *FIL* expression and miR165/166-activity were not detected. Just after a leaf primordium initiated but before the six cell layers become obvious (P1 stage, approximately 20-µm-long), most leaf cells appeared to express *FIL* and have miR165/166 activity with the exception of only a few adaxial epidermal cells (Figure S2E, S2F).

**Figure 2:**
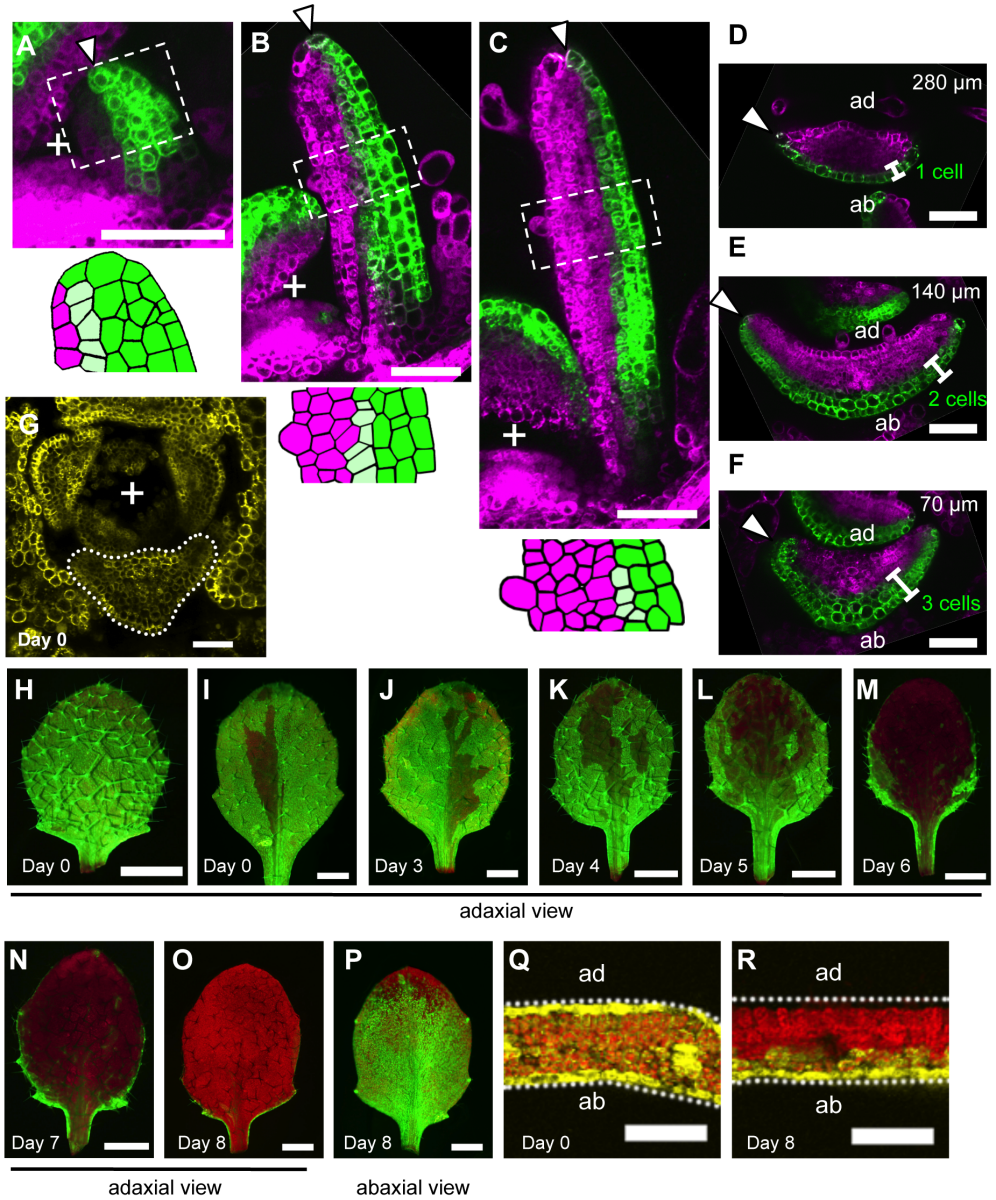
The boundary between the *FIL*-expression and miR165/166-free domains shifts during leaf development. (A–F) Confocal images of longitudinal (A–C) and transverse (D–F) sections showing *FILpro:GFP* (green) and *35Spro:miYFP-W* (magenta) marker expression patterns at different stages: 50-µm-long (A), 200-µm-long (B) and 300-µm-long (C–F). Lower schematic illustrations represent each boxed region in (A–C). (D–F), A series of sections from a leaf of approximately 300 µm in height. The approximate heights of the observation plane from the leaf base are indicated in (D–F). Arrowheads indicate the distal (A–C) and marginal (D–F) tip cells. (G–R) Confocal (G, Q, R) and stereoscopic (H–P) images showing VENUS expression patterns (yellow and yellow-green) of the *FILpro:CRE-GR 35Spro:loxP-Ter-loxP-VENUS* system in the third leaves of 12-day-old plants. The timing of DEX treatment for CRE/loxP recombination is indicated at the bottom left of each panel. The confocal imaging planes are a transverse section of a shoot apex (G) and third leaves (Q, R). The red color represents chlorophyll fluorescence. “+” marks the meristem center in all figures. Scale bars represent 50 µm (A–G), 1 mm (H–P) and 100 µm (Q, R). ad, adaxial side; ab, abaxial side.

At all of the developmental stages after the leaf initiation, the two domains were separated with not more than one-cell-width overlaps (Figure 2A–C, S2A, S2B). We hereafter refer to the boundary between *FIL-*expression and miR165/166-free domains as the FMB. In 50-µm-long primordia (P1–P2 stages), the *FIL-*expression and miR165/166-free domains occupied four to five abaxial cell layers and one to two adaxial cell layers, respectively, within the six cell layers (Figure 2A). It should be noted that *FIL* expression was detected in the distal tip cell (Figure 2A arrowhead) and the neighboring adaxial cell which presumably corresponds to future distal adaxial epidermis. Thus, FMB position was relatively adaxial rather than exactly the middle of the primordium at such early stages.

When the leaf reached 300 µm in length (P6–P7 stages), the *FIL*-expression domain was restricted to one to two cell layers in the distal part and approximately three cell layers in the proximal part of the leaf (Figure 2C–F). On the other hand, the miR165/166-free domain in this stage was expanded compared to those in the early stages keeping the slight overlap with the *FIL*-expression domain. (Figure 2C–F). Thus, FMB position was more abaxial at these stages than at the early stages. In regard to the marginal epidermis, the elongating marginal tip cells but not the neighboring adaxial cells expressed *FIL* in the distal part (arrowheads in Figure 2D, S2G–J). In contrast, the marginal tip cells and the neighboring cells still expressed *FIL* in the proximal part where marginal cell elongation is not evident yet (arrowheads in Figure 2F, S2G).

During the later stages, though the expansion of the miR165/166-free domain and restriction of the *FIL*-expression domain continued to some extent, *FIL* expression was kept in the elongating margin cells, whole abaxial epidermis and most of the abaxial-most mesophyll even after the leaves exceed 1 mm in length and the round lamina morphology is developed (Figure S2K–P). However, the GFP signal intensity of *FILpro:GFP* gradually reduced to a level not enough to distinguish *FIL*-expressing cells clearly from the other cells at further later stages (data not shown).

The FMB position changes suggest that leaf cells switch from the *FIL*-expressing state to the miR165/166-free state, and the switch occurs sequentially from the adaxial to the abaxial cell layers and also from the central to the marginal cells within a layer.

### *FIL* Expression Is Repressed Sequentially from the Adaxial Central Cells to the Abaxial Marginal Cells

Despite the clear abaxial shifting in FMB position, there remained a possibility that the switch in gene expression does not occur but gene expression states are just maintained in all cell lineages. If this were true, the FMB shifting should be attributed to rapid proliferation of the adaxial cells. To address this point by tracing the lineages of *FIL*-expressing cells, we used the dexamethasone- (DEX-) inducible CRE/loxP recombination system [41], which has recently been used *in planta* for cell lineage tracing [42], [43]. Our system consists of two constructs: *FILpro:CRE-GR* and *35Spro:loxP-Ter-loxP-VENUS*. *35Spro:loxP-Ter-loxP-VENUS* does not express *VENUS* gene without *FILpro:CRE-GR* because of the transcriptional termination sequence (*Ter*) placed between Cauliflower Mosaic Virus 35S promoter (*35Spro*) and *VENUS* regions. However, this construct can conditionally generate a marker gene, *35Spro:VENUS* (*35Spro:loxP-VENUS* in a precise description), only in the *FIL*-expressing cells by DEX-dependent and CRE-mediated DNA recombination at the two loxP sites. Thus, the *FIL*-expressing cells and their progenies are permanently marked by the fluorescence of VENUS retained in the endoplasmic reticulum after DEX application (see Text S1 and Figure S3 for the efficiency and specificity of this CRE/loxP system). If the FMB shifting is just due to the rapid proliferation of adaxial cells, DEX application at any developmental stage results in the same VENUS expression pattern. In contrast, if FMB is shifting depending on the gene expression switch, DEX applications at different developmental stages generate various expression patterns of VENUS corresponding to those of the *FILpro:GFP* at the stages of DEX application.

To reveal the *FIL*-expression dynamics during leaf development, DEX was applied to the *FILpro:CRE-GR 35Spro:loxP-Ter-loxP-VENUS* plants from a series of developmental stages and the VENUS expression patterns were observed in mature leaves. For this analysis, the third true leaves were analyzed because their growth rate is well characterized under our laboratory conditions. Fluorescent images of the adaxial-side view were captured to elucidate the quasi-three-dimensional expression patterns of VENUS in the leaves (Figure 2H–O) because the areas of strong and intermediate fluorescence intensities correspond to those of VENUS expression in the adaxial epidermis and the adaxial-most mesophyll, respectively (Figure S4). When the plants had been treated with DEX since seed germination (day 0), VENUS expression was detected either in all leaf cells or in almost all leaf cells except for a small region in the adaxial central part of the epidermis (Figure 2G–I, 2Q). DEX treatments from just before third leaf initiation (day 3) resulted in the same VENUS expression patterns as the treatments since day 0 did (Figure 2J). Given that *FIL*-expression patterns does not vary among the plants, such two patterns of VENUS expression likely reflect that *FIL* expression is initially induced in all leaf founder cells at the P0 stage and immediately repressed in the adaxial central epidermal cell(s) that does not accomplish the CRE/loxP recombination to generate the *35Spro:VENUS* gene in some cases.

DEX treatment from day 4, when the leaf reaches approximately 20- to 50-µm-long (P1–P2 stage), generated slightly more VENUS-negative cells in the adaxial central part than DEX treatment beginning on day 0 or day 3 (Figure 2K, S5E). However, the marginal epidermis still expressed VENUS in the distal and proximal parts (Figure S4A, S4D) showing a good agreement with the aforementioned *FILpro:GFP* expression pattern at the comparable stage (Figure 2A, S2E, S2F). In a series of DEX treatments beginning from day 4 to 8, the later the treatment was performed, the more the VENUS expression area was restricted to the abaxial cell layers and to lateral and proximal parts of the two adaxial cell layers (Figure 2K–O, S4, S5E). In regard to the marginal epidermis, VENUS expression is detected in the proximal part, but not in the distal part when DEX was applied from day 6 (Figure 2M, S4B, S4E, S4F). Because the third leaf reaches to 200 to 300 µm in length (approximate P6 stage) by day 6, this VENUS expression pattern is in good agreement with the *FILpro:GFP* expression pattern in such leaves (Figure 2D–F). When DEX was applied from day 8 (approximately 1,000-µm-long, P9–P10 stage), VENUS expression in the two adaxial cell layers almost disappeared (Figure 2O, S4C, S5E), but two or three abaxial cell layers still expressed VENUS (Figure 2P, 2R, S4G, S4H), showing a good agreement again with the *FILpro:GFP* expression pattern (Figure S2K–P). In summary, the series of DEX treatments generated a variety of the VENUS expression patterns each of which highly correlates with each *FILpro:GFP* expression pattern at the corresponding stage. Therefore, it is indicated that FMB shifting is mainly caused by the gene expression switch from the *FIL*-expressing to the non-*FIL*-expressing state in each cell.

### A Mutant *enf2* Shows Slow FMB Shifting

**Figure 3:**
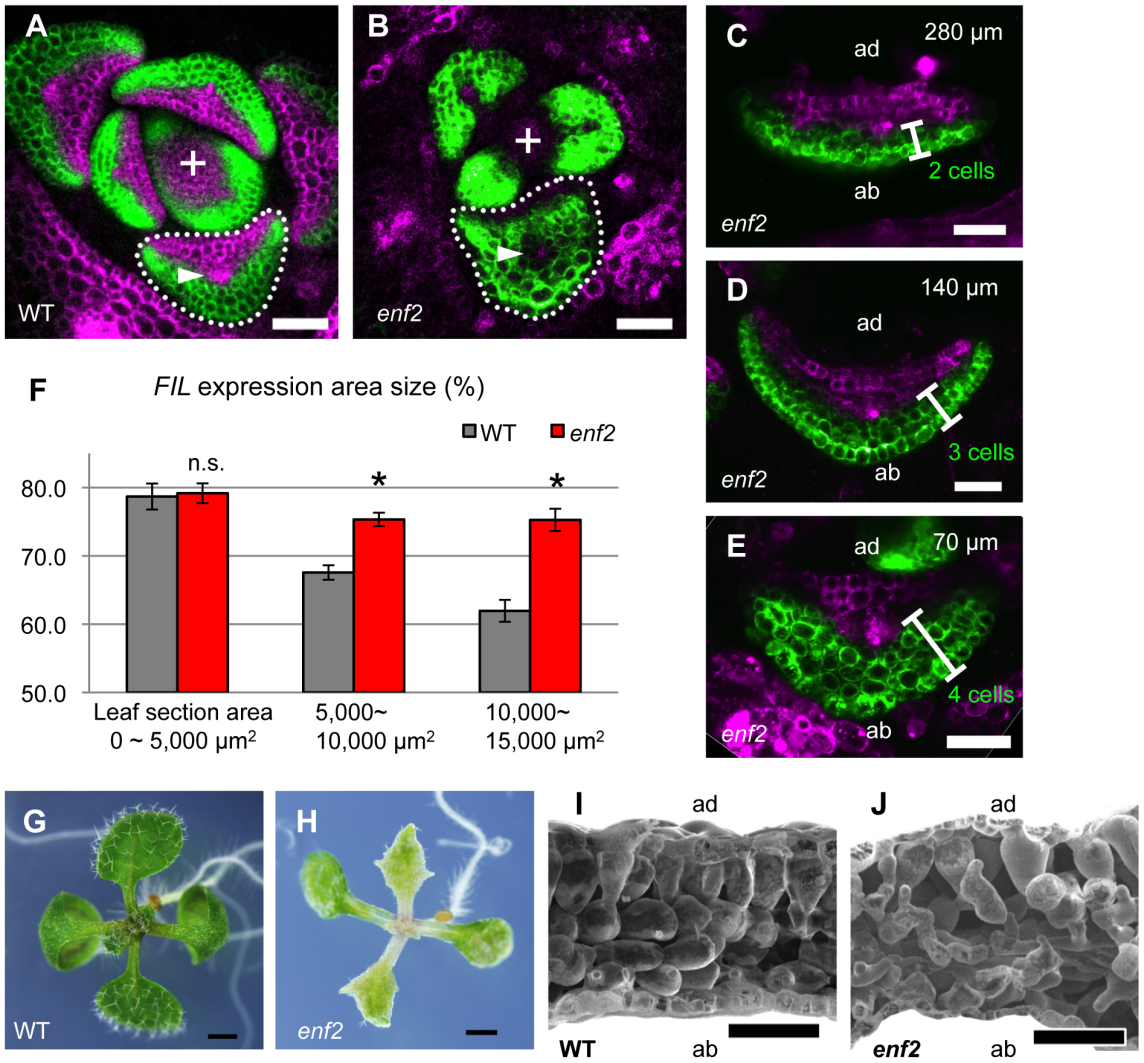
The *enf2* mutant shows slow FMB shifting and an abaxialized leaf phenotype. (A–E) Confocal images of transverse sections showing *FILpro:GFP* (green) and *35Spro:miYFP-W* (magenta) marker expression in the wild-type (A) and *enf2* (B–E) leaf primordia. The arrowheads indicate the provascular cells. (C–E), A series of sections from a leaf of approximately 300 µm in height. The approximate heights of the observation plane from the leaf base are indicated. The comparable WT data are Figure 2D–F. (F) *FIL*-expression area sizes (%, y-axis) at different stages (grouped by section area sizes, x-axis) of the wild-type and *enf2* leaf primordia. Bars indicate standard errors. n.s., not significantly different; *, significantly different (*p*<0.05, t-test) between the wild type and *enf2*. (G, H) Seedlings of the wild type and *enf2*. (I, J) Scanning electron microscope images of leaf sections from the wild type and *enf2*. Scale bars represent 50 µm (A–E, I, J) and 1 mm (G, H). WT, wild type.

To gain insights into the regulatory mechanisms for FMB shifting, we sought mutants defective in the shifting process. A candidate of such mutants, *enlarged fil expression domain2* (*enf2*) was isolated from a genetic screen for altered *FILpro:GFP* expression patterns [29]. Whereas the *FIL*-expression domain in the wild type did not surround the center provascular cells (Figure 3A), the domain in this mutant was abnormally large, frequently surrounding the provascular cells in around the P5 stage (Figure 3B).

To characterize the dynamics of *FIL*-expression and miR165/166-free domains, the markers *FILpro:GFP* and *35Spro:miYFP-W* were observed in various developmental stages of *enf2* leaf primordia. Whereas the two domains were almost separated at all stages in this mutant as in the wild type, the domain sizes were different between *enf2* and the wild type especially in later developmental stages (Figure 3A–E, S5A–D). In small leaf primordia, the size of the *FIL*-expression domain in *enf2* was comparable to that in the wild type (Figure 3F, S5A, S5C). By contrast, in relatively large leaf primordia, the *FIL*-expression domain in *enf2* was significantly larger than that in the wild type (Figure 3F, S5B, S5D). For example, in 300-µm-long primordia of *enf2*, the *FIL*-expression domain was restricted to two to four abaxial cell layers (Figure 3C–E). Such a domain size was larger than that in the wild type at the same developmental stage (Figure 2D–F, 3C–F) suggesting the slower FMB shifting than in the wild type. Nonetheless, the *FIL*-expression domain in *enf2* at this stage was smaller than that in the early stages (Figure S5C, S5D), suggesting that the FMB shifting did occur even in this mutant. Furthermore, the *FILpro:CRE-GR* and *35Spro:loxP-Ter-loxP-VENUS* system revealed that *FIL* expression is initially induced in all of the leaf cells and gradually restricted during later stages in *enf2* (Figure S5F–N). This analysis also showed that the sizes of the VENUS-negative area in leaves treated with DEX from day 3 did not significantly differ between the wild type and *enf2* (the left data points in Figure S5E). This suggests that the earliest repression of *FIL* expression in the adaxial central epidermis at the P0 stage is not affected in *enf2*. The results from marker observation and lineage tracing can be interpreted as showing that the FMB shifting occurs in *enf2*, but more slowly than in the wild type.

### Altered FMB Shifting Is Associated with Defects in the Lamina Expansion and the Adaxial-Abaxial Cell Differentiation

Mature leaves in *enf2* are pale green, more serrated and narrower than those in the wild type (Figure 3G, 3H). In some cases, *enf2* formed needle-like leaves lacking trichomes at a frequency of less than one percent (Figure S5O). These narrow and needle-like morphologies are similar to those of abaxialized leaves, including the leaves of *35Spro:FIL* plants [13], [14], *rev* recessive mutants harboring another enhancer mutations [10], [44], [45] and *35Spro:MIR165* plants [46]–[48]. Further observations of leaf anatomy by scanning electron microscopy revealed that adaxial mesophyll cells in *enf2* were not as densely packed and columnar shape as those of the wild type but had air space and a bumpy cell surface looking more like that of the abaxial spongy mesophyll of the wild type (Figure 3I, 3J). In summary, *enf2* leaves are partially abaxialized with respect to the lamina morphology and mesophyll differentiation, suggesting that slow FMB shifting results in narrow lamina formation and abaxialized mesophyll.

**Figure 4:**
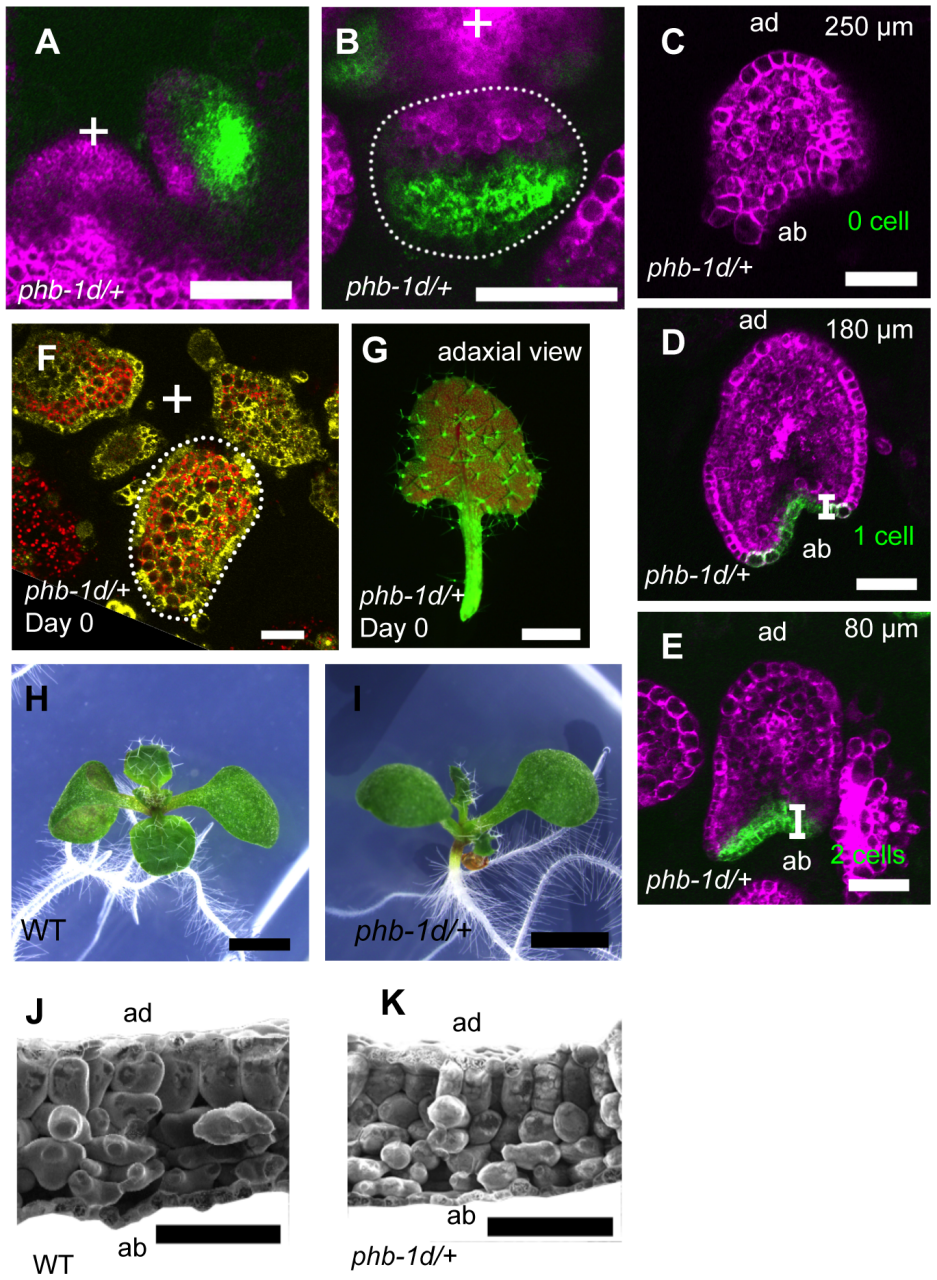
FMB shifting is quicker in the *phb-1d/+* mutant than in the wild type. (A–E) Confocal images of longitudinal (A) and transverse (B–E) sections showing *FILpro:GFP* (green) and *35Spro:miYFP-W* (magenta) marker expression in *phb-1d/+* leaf primordia. (C–E), A series of transverse sections from a leaf of approximately 300 µm in height. The comparable WT data are Figure 2D–F. (F, G) VENUS expression pattern (yellow and yellow-green) of *FILpro:CRE-GR 35Spro:loxP-Ter-loxP-VENUS* in *phb-1d/+*. A transverse section of leaf primordia (F) and a stereoscopic image of mature leaf (G). (H, I) Seedlings of the wild type and *phb-1d/+*. (J, K) Scanning electron microscope images of leaf sections in the wild type and *phb-1d/+*. Scale bars represent 50 µm (A–F), 1 mm (G–I) and 100 µm (J, K).

To know the relationship between the leaf morphological features and the FMB shifting speed, we analyzed FMB shifting in *phb-1d* heterozygous (*phb-1d/+*) plant, which is well known for the partially adaxialized leaf phenotype of narrow and cup-shaped leaves [49] (Figure 4H, 4I) with excessive densely packed and smooth mesophyll cells [50] (Figure 4J, 4K). When *FILpro:GFP 35Spro:miYFP-W* markers were introduced into *phb-1d/+*, the *FIL*-expression and miR165/166-free domains were separated as in the wild type (Figure 4A–E). The *FIL*-expression domain occupied three to four abaxial cell layers in the early primordium (approximately 50-µm-long) (Figure 4A, 4B; see also Figure 2A, S5A) and two to zero cell layers in the later primordium (approximately 300-µm-long) (Figure 4C–E; see also Figure 2D–F). The *FILpro:CRE-GR* and *35Spro:loxP-Ter-loxP-VENUS* system revealed that *FIL* is expressed throughout the leaf at the initiating stages, even in *phb-1d/+* plants (Figure 4F, 4G). Therefore, the speed of FMB shifting is greater in *phb-1d/+* than in the wild type, suggesting that fast FMB shifting results in narrow and abnormal lamina formation and excess adaxialization of mesophyll.

### *ENF2* Encodes a Plastid-Localized Protein

**Figure 5:**
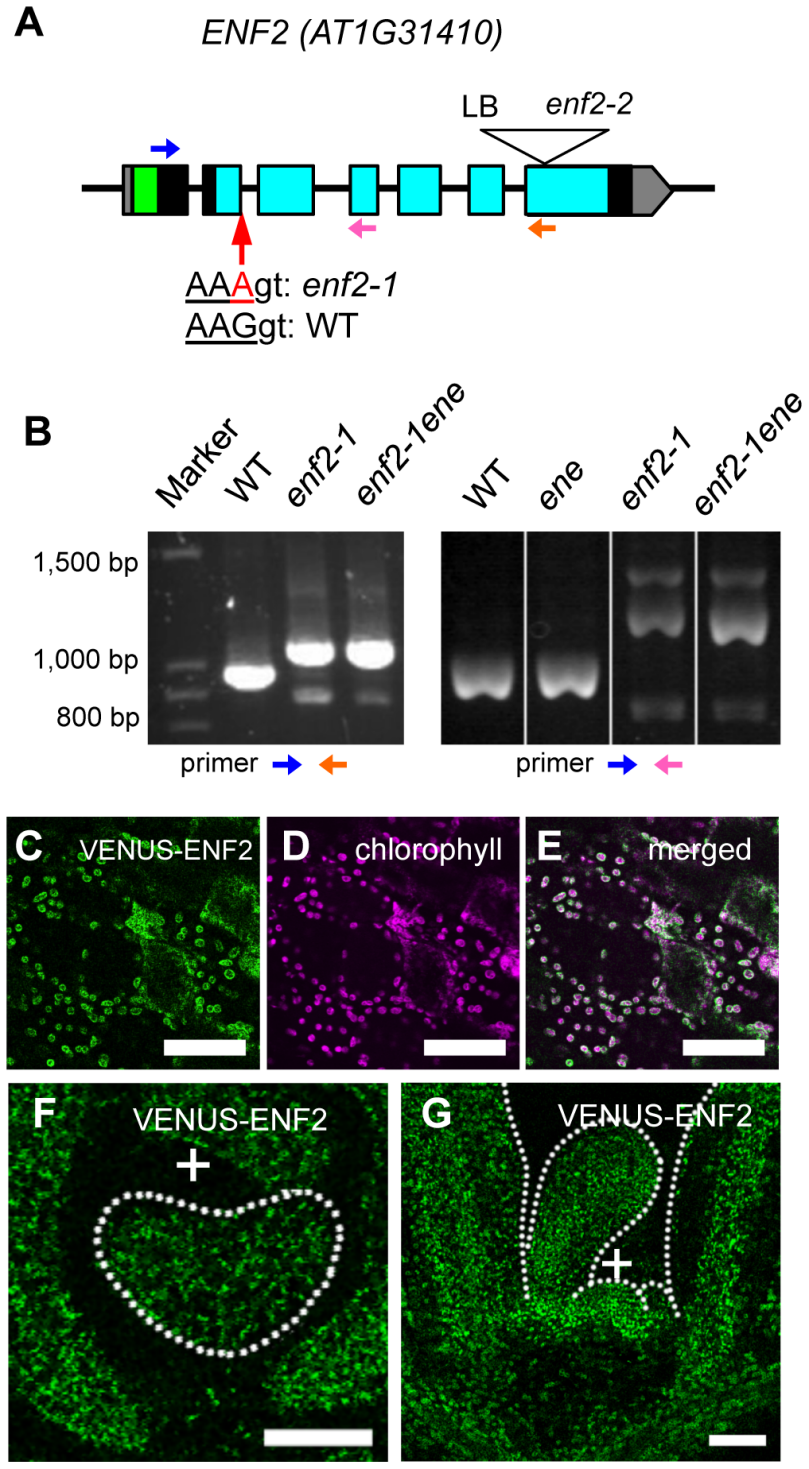
The *ENF2* gene encodes a plastid-targeted protein expressed throughout the shoot apex. (A) Schematic representation of the *ENF2* gene. Boxes represent exons. The untranslated regions, the putative transit peptide and the PotD/F homology domain are highlighted in gray, green and blue, respectively. The mutations in the *enf2-1* and *enf2-2* alleles are indicated. LB, left boarder of T-DNA. Blue, pink and orange arrows indicate the primers for RT-PCR analysis (B). (B) RT-PCR using the primers represented as the blue and orange arrows (left), the blue and pink arrows (right). (C–E) Confocal images showing VENUS-ENF2 (green, C), chlorophyll (magenta, D) fluorescence and both (E) in petiole cells. (F, G) Confocal images showing a transverse (F) and longitudinal (G) sections of a shoot apex expressing *ENF2pro:VENUS-ENF2* (green). Scale bars represent 50 µm (C–G).

A positional cloning approach found a single base substitution at the second exon terminus (*enf2-1* allele) of the gene *AT1G31410* in the *enf2* genome (Figure 5A). Because the mutant phenotype was rescued by introducing the wild-type genomic fragment of this gene (Figure S6A–C), we concluded that *ENF2* is *AT1G31410*. The *enf2-1* mutation results in no amino acid substitution when the mRNA is spliced as in the wild type. However, RT-PCR analysis revealed that the mutation leads to unusual splicing events (Figure 5B) that generate premature stop codons in the majority of the mRNA (data not shown). Thus, the amount of functional *ENF2* mRNA is reduced in this mutant. However, the mutant of another *enf2* allele (*enf2-2*) found in the SALK T-DNA insertion lines (Figure 5A) showed whiter and narrower leaves than *enf2-1* and was seedling lethal (Figure S7B). In addition, *ENF2* mRNA with the normal exon junctions was detected from the *enf2-1* mutant as a small peak in an electropherogram of the RT-PCR product (Figure S7A). Therefore, viable *enf2-1* mutant retains some *ENF2* function and is a weak allele that is useful for analyses of *ENF2* function without side effects of lethality.

We found an *enf2* enhancer mutation (*ene* hereafter) in the process of cloning *ENF2*. The existence of this enhancer locus was indicated by the fact that some F2 plants from the cross between the wild type and *enf2* showed a milder mutant phenotype than the parental *enf2* in terms of the leaf morphology, color (Figure S7D, S7F) and *FIL*-expression pattern (Figure S7G). The positional cloning approach and rescue experiments identified a missense mutation in *AT1G80070/SUS2* as the *ene* mutation (Figure S7H–L). This gene product is a homolog of yeast Prp8, which plays an important role in recognizing exon-intron junctions during mRNA splicing [51]. To examine the possibility that the *ene* mutation weakens *ENF2* function by affecting the splicing efficiency of *ENF2* mRNA in the *enf2-1* mutant, we compared the amounts of normally spliced *ENF2* mRNA in the *enf2-1* single mutant and the *enf2-1 ene* double mutant. The electropherogram of the *ENF2* RT-PCR products revealed that *enf2-1 ene* contained less wild-type mRNA than *enf2-1* (Figure S7A). Moreover, the *ene* single mutant was indistinguishable from the wild type in leaf morphology, color (Figure S7C, S7E) and *FIL*-expression pattern (Figure S7G). These data suggest that the *ene* mutation enhances the *enf2* phenotype by decreasing the splicing efficiency of the *enf2-1* allele, and we continued to use the *enf2-1 ene* double mutant (described as *enf2* again below) for further analyses as a plant defective in *ENF2* function.

To analyze expression and subcellular localization of ENF2 protein, we created transgenic plants expressing *VENUS-ENF2* fusion gene under the control of the *ENF2* promoter. This transgene, *ENF2pro:VENUS-ENF2*, was able to rescue *enf2* (Figure S6A, S6B, S6D), indicating that *ENF2pro:VENUS-ENF2* can confer authentic *ENF2* expression and that VENUS-ENF2 is functional. VENUS-ENF2 was localized in chloroplasts (Figure 5C–E), suggesting that ENF2 is localized in chloroplasts. This result is consistent with recent reports of proteomic analyses that the *Arabidopsis* ENF2 protein and its maize homolog were detected in the plastid (chloroplast) fraction [52], [53]. To clarify ENF2 function, the *ENF2pro:VENUS-ENF2* expression pattern was analyzed. VENUS-ENF2 was expressed throughout the shoot apical meristem and leaf primordium (Figure 5F, 5G). Such a broad expression pattern of *ENF2* is consistent with a previous transcriptomic study in which the *ENF2* mRNA was detected from the *FIL*-expressing part and the central-zone of the shoot apical meristem [54] (http://bar.utoronto.ca/efp/cgi-bin/efpWeb.cgi).

While there is no previous report showing the protein function of ENF2 and the homologs in plant, BLAST search (protein blast in NCBI: http://blast.ncbi.nlm.nih.gov/Blast.cgi) found that ENF2 amino acid sequence shows low similarity to those of two bacterial proteins, PotD (44% similarity) and PotF (46% similarity), which specifically bind to polyamines and transport them into cells [55]. The amino acid residues indispensable for the polyamine binding of PotD and PotF are widely conserved among the bacterial homologs [55] (Figure S8 red). However, the corresponding regions are substituted in ENF2 by dissimilar residues that are conserved only among green plant lineages and cyanobacteria (Figure S8 green). The similarity and difference among the homologous proteins might imply that ENF2 in plastids has some functions similar to but distinct from those of PotD and PotF in bacteria.

### ENF2 Is Required for Chloroplast Development and Plastid Gene Expression in Leaf Primordia

The mutant phenotypes of pale green (*enf2*) (Figure 3H) and white (*enf2-2*) (Figure S7B) color and the localization of ENF2 protein to plastid imply a role of *ENF2* in chloroplast development. To characterize the effect of the *enf2* mutation on chloroplast development, we observed the plastid inner structures by transmission electron microscopy (Figure S9A–F). Mature *enf2* leaves had normal-looking chloroplasts because they showed highly stacked thylakoid membranes and large starch granules, as did the wild-type leaves (Figure S9C, S9F). In contrast, plastids in the *enf2* leaf primordia had less-developed inner structures than those in the wild type in comparable stages (Figure S9B, S9E). Taken together with the similarity of the proplastid structure in the meristems of wild type and *enf2* (Figure S9A, S9D), the chloroplast development is likely to be delayed in the mutant.

The *enf2* phenotypes of pale green appearance, narrow laminae and defective differentiation of palisade mesophyll are common defects to plants harboring dysfunctional plastidial ribosome or plastidial RNA polymerase [56]–[63]. To examine whether plastid gene expression is impaired in *enf2*, the expression levels of all of the 80 protein-coding and two rRNA genes encoded by the plastid genome were analyzed by Quantitative Reverse Transcription PCR (qRT-PCR). On average, the expression levels were reduced to 49.0% of the wild type, with a range of 93.2% to 23.7% (Figure S9G). Because the amounts of rRNA were also reduced (the right-most two bars in Figure S9G), plastid gene expression might be globally down-regulated, not only at the RNA level but also at the level of translation in *enf2*.

It has been known that the plastid gene expression profile is affected when chloroplast development is inhibited by external stresses [64] and that inhibition of plastid gene expression by chemical treatments or genetic mutations leads to defective chloroplast development [59], [61], [65], [66]. ENF2 is also important for chloroplast development and plastid gene expression, though it is unclear which of the chloroplast development and plastid gene expression is primarily affected in *enf2*.

### Inhibition of Plastid Gene Expression Leads to Retardation of FMB Shifting

The abnormalities in plastid condition suggest the involvement of plastid function in regulating the FMB shifting. To test whether the defective chloroplast development is sufficient to retards FMB shifting and lead to narrow lamina development, chloroplast development was inhibited in the wild type by norflurazon treatment and dark growth condition. Norflurazon is an inhibitor of carotenoid biosynthesis [67] that bleaches seedlings by causing oxidative damaging of plastids in the light. Although application of 0.25 µM norflurazon caused leaf bleaching, the leaves had roundly expanded lamina even at 25 µM concentration (Figure S10A). Dark-grown seedlings were etiolated and lacking in chloroplast but formed round lamina (Figure S10B). The narrow appearance of the leaves was due to their elongated petioles (Figure S10C). Neither norflurazon nor dark condition led to as clear alterations in the *FILpro:GFP 35Spro:miYFP-W* expression patterns (Figure S10D, S10E) as *enf2* showed. These results indicate that the FMB shifting and lamina morphology are not necessarily affected by the inhibition of chloroplast development.

**Figure 6:**
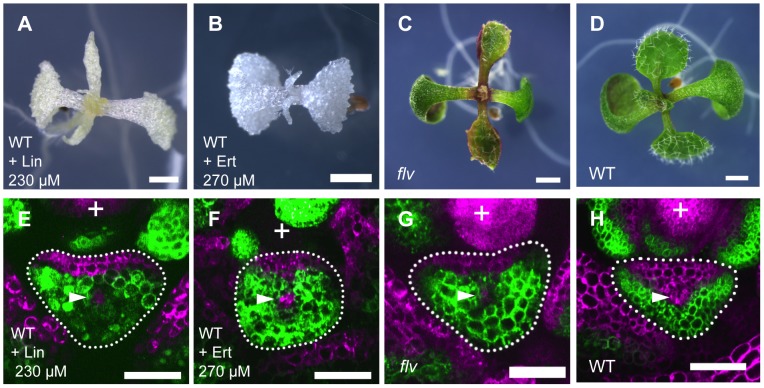
Inhibition of plastid gene expression machinery retards FMB shifting and leads to narrow lamina formation. (A–D) Seedlings of lincomycin- and erythromycin- treated wild type, and untreated *flv* mutant and wild type. (E–H) Confocal images of transverse sections showing *FILpro:GFP* (green) and *35Spro:miYFP-W* (magenta) marker expression in leaf primordium of each above plant. The arrowheads indicate the provascular cells. Scale bars represent 1 mm (A–D) and 50 µm (E–H). Lin, lincomycin; Ert, erythromycin.

To know whether the plastid gene expression activity affects the FMB shifting and lamina development, we inhibited the plastid translation in the wild type by lincomycin and erythromycin treatments. Both lincomycin and erythromycin specifically inhibit plastidial ribosome without clear effects on cytosolic and mitochondrial ribosomes [68]. At concentrations of 100 µM, these chemicals bleached seedlings, and narrow and filamentous leaf formation was observed at concentrations greater than 200 µM (Figure 6A, 6B). In the leaf primordia of the lincomycin- and erythromycin-treated plants, *FILpro:GFP 35Spro:miYFP-W* marker expression patterns showed that the FMB positions were close to the adaxial side, similarly to that in *enf2* (Figure 6E, 6F). The effects of lincomycin and erythromycin treatments on the lamina morphology and FMB position indicate that *enf2*-like phenotype is regenerated by inhibition of protein synthesis in plastids.

Though the lincomycin- and erythromycin-treated plants showed *enf2*-like phenotype in morphology, they were albino and seedling lethal suggesting that plastid function is more extensively affected in these plants than in *enf2* which is pale green and viable. To examine whether the impaired plastid gene expression is responsible for defective FMB shifting independently of abnormal photosynthesis and developmental arrest, we sought a mutant impaired in plastid gene expression machinery that exhibits narrow lamina with a mild phenotype in color and viability. For this purpose, the mutant *flavodentata* (*flv*) was selected because the *FLV* gene has been shown to encode a plastid-localized PPR protein required for RNA editing of the *rpoC1* mRNA, which encodes a subunit of plastid-encoded RNA polymerase (I. Small, personal communication), and because *flv*, which is allelic to *defectively organized tributaries4* [69] (I. Small, personal communication), is viable and known for its narrow serrated leaves that are pale green in color [69], [70] (Figure 6C). Observations of *FILpro:GFP 35Spro:miYFP-W* marker expression in *flv* revealed that the FMB position was close to the adaxial side, similarly to that of *enf2* (Figure 6G). This result supports the role of plastid gene expression in FMB shifting and lamina expansion, and shows the separable nature of this role from the development of the photosynthetic apparatus and lethality.

From the result that *enf2*-like phenotype was regenerated by lincomycin and erythromycin treatments and *flv* mutation but not by norflurazon treatment and dark growth condition, it is suggested that the impaired plastid gene expression is the key to the defective FMB shifting and lamina morphology in *enf2*. However, it has been known that the plastid gene expression profile of the whole seedling RNA is also globally fluctuated by norflurazon treatment [71], . To examine whether or not the alterations in plastid gene expression in the shoot apex is less severe in the norflurazon-treated plants than in *enf2*, we also quantified the expression levels of several plastid genes in these plants. The result showed that not only the photosynthesis-related genes (*rbcL*, *psaA*, *psaB*, *psbA*, *psbE*) but also the genes for transcription (*rpoA*, *rpoB*, *rpoC1*, *rpoC2*) and for translation (*rrn16S*, *rrn23S*) are down-regulated as severely as or more than in *enf2* (Figure S10F). This means that the expression levels of plastid genes in the whole shoot apex tissue are not always linked to the *enf2*-like phenotype. Nonetheless, simultaneous treatment of norflurazon and lincomycin resulted in narrow lamina formation (Figure S10G). This result indicates that the norflurazon-treated plants are not insensitive to, but show the developmental response to, the inhibition of plastid gene expression. Therefore, it is likely that the *enf2*-like phenotype is caused by direct inhibition of plastid gene expression machinery rather than by the reduced levels of plastid gene expression.

To further characterize the similarity between *enf2* mutation and the lincomycin treatment, *enf2* was also treated with lincomycin. When *enf2* was treated with 150 µM of lincomycin, filamentous leaves were formed in more than 90 percent of seedlings (Figure S10H). In contrast to *enf2*, wild type required 450 µM of lincomycin to form such leaves at a similar frequency (Figure S10I). This result supports that *enf2* mutation leads partial impairment in plastid gene expression as a certain concentration of lincomycin does, thus they both retard FMB shifting and inhibit lamina expansion.

### The Retardation of FMB Shifting by Plastid Depends on the *GUN1*-dependent Retrograde Signal

The mechanism with which nuclear gene expression levels respond to the plastid condition is called the plastid retrograde signal. It has been known that changes in plastid gene expression and other plastid conditions affect the expression levels of nuclear genes, including photosynthetic genes, through the *GENOMES UNCOUPLED1*- (*GUN1*-) dependent retrograde signal (see [36]–[40] for review). In addition, another specific retrograde signal is also known to couple the nuclear gene expression with tetrapyrrole biosynthetic activity but not with plastid gene expression. Other *GUN* genes (*GUN2*, *3*, *4* and *5*) are involved in this specific pathway [73], [74].

**Figure 7:**
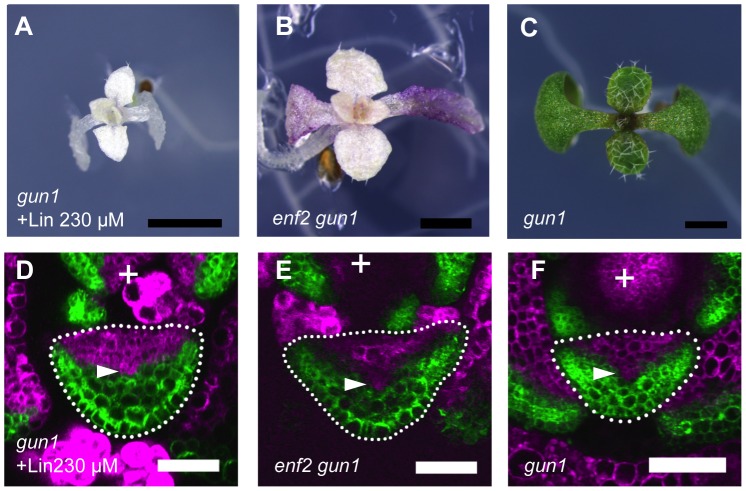
The plastid effects on FMB shifting and lamina expansion depend on the *GUN1* gene. (A–C) Seedlings of lincomycin-treated *gun1*, untreated *enf2 gun1* and untreated *gun1*. (D–F) Confocal images of transverse sections showing *FILpro:GFP* (green) and *35Spro:miYFP-W* (magenta) marker expression in leaf primordium of each above plant. The arrowheads indicate the provascular cells. Scale bars represent 1 mm (A–C) and 50 µm (D–F).

To examine the involvement of known retrograde signals in the plastid effect on the lamina expansion and FMB shifting, we analyzed the responses of *gun* mutants to the inhibition of plastid gene expression. While *gun2*, *3*, *4* and *5* mutants formed narrow or filamentous leaves as the wild type did (Figure S11A and data not shown), the *gun1* mutant formed relatively round lamina (Figure 7A) when they were treated with lincomycin. The lincomycin-treated *gun1* showed a similar FMB position to that of the untreated wild type (Figure 7D), suggesting that the unaffected FMB position in leaf primordia is the basis for the lamina expansion. In contrast to such distinct phenotype of *gun1* under the lincomycin-treated condition, untreated *gun1* mutant showed the leaf morphology indistinguishable from that of the wild type (Figure 7C), as previously reported [75], [76]. The FMB position in *gun1* leaf primordia did not differ significantly from that of the wild type at any stage of leaf development (Figure 7F, S11C). These *gun1* phenotypes indicate that the *GUN1* is involved in the retardation of FMB shifting and defective lamina expansion only in response to the inhibition of plastid gene expression, but not under normal plastid conditions.

If the retarded FMB shifting in *enf2* is due to the failure of plastid gene expression, additional *gun1* mutation may suppress this phenotype. As expected, the *enf2 gun1* double mutant, but not *enf2 gun5*, showed round lamina (Figure 7B, S11B). The FMB position in *enf2 gun1* was similar to that of the wild type grown normally (Figure 7E, 6H). However, *enf2 gun1* was different from *enf2* not only in the FMB position and the lamina morphology, but also in the color and viability. The double mutant showed an almost albino phenotype and was seedling lethal (Figure 7B), suggesting that the suppressed developmental phenotypes were not due to rescued plastid condition. To confirm this point, we checked plastid gene expression levels in *enf2 gun1* by qRT-PCR. Though the expression levels of the transcription-related genes (*rpoA*, *rpoB*, *rpoC1*, *rpoC2*) were similar to those in the wild type, the amount of ribosomal RNA (*rrn16S*, *rrn23S*) and other genes' mRNA were much more reduced in *enf2 gun1* than in *enf2* (Figure S11D). Therefore, it is likely that that *gun1* mutation suppresses the *enf2* phenotype not by rescuing the failure of plastid gene expression, but by diminishing the response to the abnormal plastid condition.

The albino and lethal phenotype of *enf2 gun1* and the pale green and viable phenotype of *enf2* suggest that the wild type allele of *GUN1* is important for the viability and autotrophic growth of the plant when the plastid gene expression is accidentally impaired. This hypothesis was also supported by the observation that the wild type and *gun1* plants differed in the viability after transient lincomycin treatment. After transfer from the lincomycin-containing medium to the standard medium, wild type plants produced green shoots and continued growth, whereas *gun1* did not grow any longer (Figure S11E, S11F). These results indicate that *GUN1* has a role in retarding FMB shifting and inhibiting lamina expansion when plastid gene expression is inhibited by internal and external damages, but the plant can adapt to such a severe situation thanks to the *GUN1* function.

## Discussion

In this study, we revealed the following two points.

**Figure 8:**
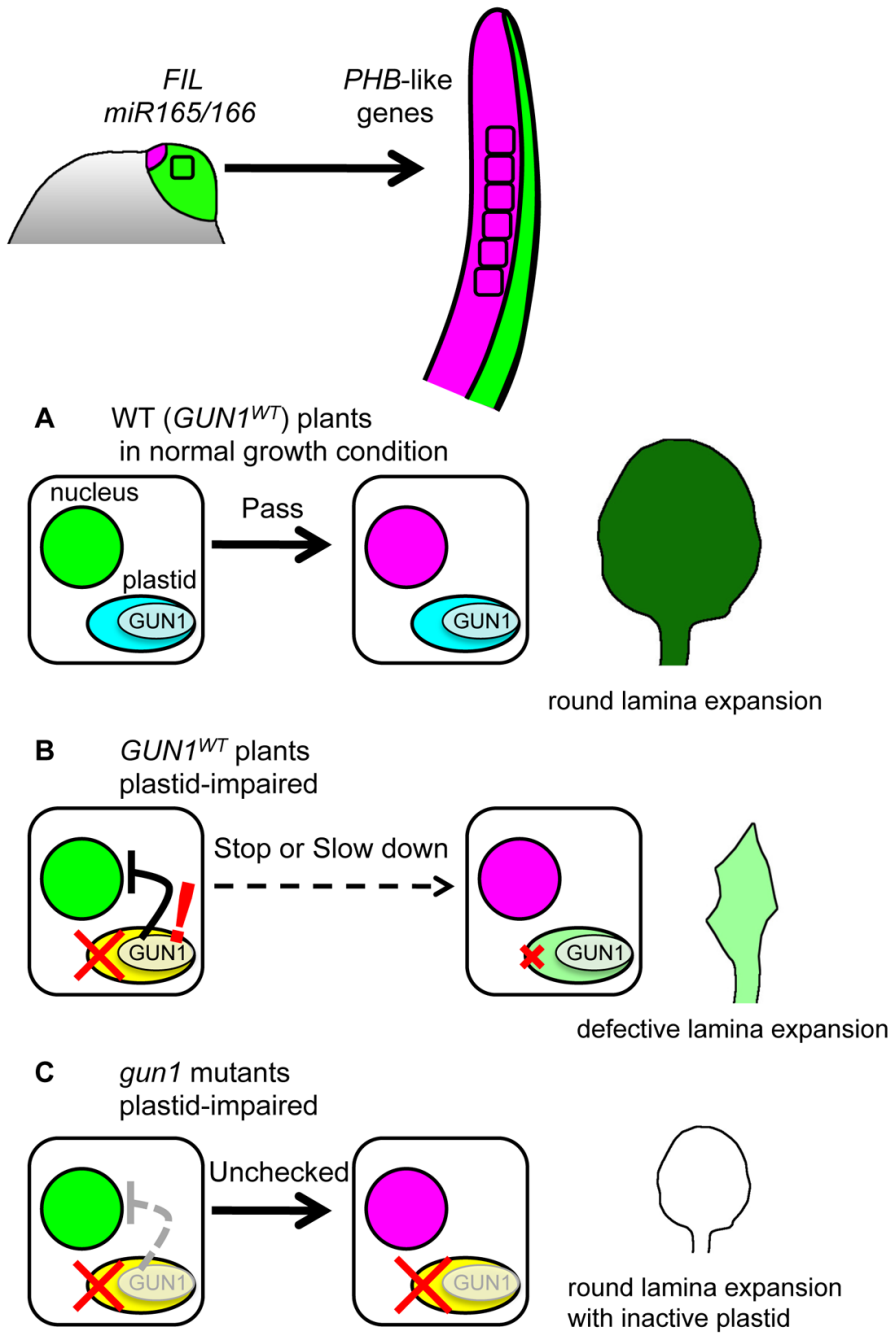
Model for FMB regulation by GUN1-dependent retrograde signal. Most leaf cells express *FIL* and have miR165/166 activity just after leaf initiation. However, during the early developmental stages, the *FIL*-expressing and miR165/166-active cells switch the nuclear gene expression state to that expressing *PHB*-like genes, thus FMB shifts. When plastid gene expression machinery is functional (A), the gene expression switch in nuclei progress smoothly regardless whether GUN1 is functional or not. The pace of this gene expression switch is important for the full lamina expansion. When the plastid gene expression machinery is impaired (B), the *GUN1*-dependent retrograde signal affects the nuclei to delay or stop the gene expression switch. This plastid effect contributes to prevent the wide lamina expansion. Possibly, the *GUN1*-dependent retrograde signal regulates also other nuclear genes to repair the plastid condition. When the plastid gene expression machinery is impaired and the plant is devoid of the *GUN1*-dependent retrograde signal (C), the switch in nuclear gene expression progress normally and lamina expands despite the absence of photosynthetic activity.

1. The boundary between *FIL* expression and miR165/166-free domains shifts from adaxial side to the abaxial side during leaf primordial development. This boundary shifting is caused by alternative transfer of cellular gene expression profile from the abaxial-region specific to the adaxial-region specific (Figure 8A). Altered speeds of the boundary shifting are associated with the morphological changes in lamina expansion and in mesophyll differentiation.
2. When plastid condition is impaired by chemical treatments and genetic mutations affecting plastidial ribosome and RNA polymerase, the boundary shifting is retarded and the lamina morphology becomes narrow (Figure 8B). These plastid effects depend on *GUN1* gene (Figure 8C).

These points together highlight a sophisticated developmental regulation to ensure the total photosynthetic efficiency of leaves by preventing the lamina expansion with inactive plastids. The molecular mechanisms and physiological importance of each point are discussed below.

### The Mechanism and Developmental Importance of FMB Shifting

The mathematical model shows that a boundary between two gene expression domains easily shifts when the genes repress each other's expression via mobile factors. Though the whole regulatory network for the adaxial- and abaxial- specific genes is still largely unclear [25], this theory of boundary shifting gives a good working hypothesis for the mechanism of FMB shifting because of the following reasons. The mathematical model assumes only three points; two (groups of) genes (*AD* and *AB*) are repressing each other's expression; a cell can express only one (group) of the genes due to the mutual repression when there is no external cues fluctuating the gene expression; some of the gene products move between neighboring cells depending on the concentration gradient. Accordingly, a mutual repressive relationship is known at least between *PHB*-like genes and miR165/166 whereas the regulatory relationship between *PHB*-like genes and *FIL* is yet to be elucidated. *PHB*-like genes repress miR165/166 activity by positively regulating *AGO10*/*PINHEAD* [77], [78], by decreasing miR165/166 expression level via cytokinin signal [79] and possibly by activating tasiR-ARFs [25]. The activity of miR165/166 in turn represses the expression of *PHB*-like genes through mRNA cleavage [80] and DNA methylation [81]. In this context, the candidates of the mobile factors are miR165/166, cytokinin and possibly tasiR-ARFs. Therefore, an important suggestion from the mathematical model is that any unknown factors are not necessarily required to explain the shifting nature of FMB. Our mathematical model is also compatible with the *phb-1d/+* phenotype because the shifting speed of the domain boundary toward the abaxial side is increased in the computer simulation when the *AD* degradation by *AB* is weakened (Figure S12). Such situation roughly corresponds to *phb-1d*/+ mutant in which the cleavage efficiency for *PHB* mRNA by miR165/166 is reduced [80] and the speed of FMB shifting is faster than in the wild type. Further comparisons between the model and real observations will be an important approach to evaluate the model and elucidate the molecular basis for the FMB shifting.

Our data suggest that the speed of the FMB shifting is important for round and wide lamina expansion (Figure 8A) because fast and slow FMB shifting were associated with narrow or abnormal lamina formation in *phb-1d/+* and *enf2* mutants. It has been characterized well that the functions of *FIL*, *PHB*-like genes and miR165/166 are required for the lamina growth because their loss-of-function mutations and overexpression lead to narrow lamina or needle-like leaf formation [10], [13], [14], [44]–[48]. However, a clear explanation of how FMB shifting is linked to the lamina expansion is one of the future challenges. Lamina expansion in the lateral direction requires *WUSCHEL-RELATED HOMEOBOX1* (*WOX1*) and *PRESSED FLOWER/WOX3* (*PRS*) expression [34] and local auxin biosynthesis by *YUCCA* genes [82] at the adaxial-abaxial juxtaposition domain. Therefore, the expression domains and durations of such genes for lamina expansion might be regulated in response to the stage-specific positions of FMB. To further characterize the relationship between FMB shifting and lamina expansion, an important challenges is the three-dimensional live-imaging of FMB shifting with simultaneous monitoring of cell proliferation and other genes' expression.

### The Mechanism of the FMB Position Regulation through *GUN1*-Dependent Retrograde Signal

Our data show that chemical and genetic inhibition of plastid gene expression machinery retards FMB shifting via the *GUN1*-dependent mechanism (Figure 8B). It is known that the *GUN1*-dependent retrograde signal down-regulates photosynthetic genes in the nucleus by changing the expression levels of the transcription factor genes *ABI4* and *GLK1*, which subsequently changes the expression levels of the downstream photosynthetic genes [73], [83], [84]. One possible scenario is that *ABI4* and *GLK1* also affect the expression levels of *FIL*, *miR165/166* and other adaxial- and abaxial-specific genes through the transcriptional regulation. Among the adaxial- and abaxial-specific genes, *KAN1* and *ETTIN*/*ARF3* are known to be up-regulated in response to the impaired plastid gene expression though the involvement of *GUN1* in the up-regulation is unclear [62]. On the other hand, it is also possible that the slow FMB shifting is a more indirect effect than such direct transcriptional regulations. For example, there are some reports pointing out that lincomycin-treated plant differently express the genes encoding cytosolic ribosomal proteins [85] and that some mutants of the genes for cytosolic ribosomal proteins form the partially abaxialized leaves as plastid-defective mutants do [62], [86]–[89]. Another study shows the importance of abscisic acid metabolism for the leaf morphological phenotype of a plastid-defective mutant [63]. These reports suggest a possibility that changes in the cytosolic ribosomal proteins and abscisic acid metabolism mediate the regulation of the FMB position by the *GUN1*-dependent retrograde signal.

### The Physiological Importance of the Leaf Developmental Regulation through *GUN1*-Dependent Retrograde Signal

Previous genetic studies have reported that the mutant plants harboring dysfunctional plastidial ribosomes or plastidial RNA polymerase show narrow laminae and/or defective palisade mesophyll differentiation as *enf2* does [56]–[63]. However, the question of whether such developmental effects reflect only the inability to run the normal developmental program or a significant response to the plastid dysfunction has been unanswered. This question is partially answered by the phenotype of *enf2 gun1* and lincomycin-treated *gun1* because their normal FMB positions and round laminae indicate that the leaf primordia retain the ability to run the developmental program for FMB shifting and lamina expansion even when the plastids are dysfunctional (Figure 8C). This finding raises a question of whether the inhibition of such normal lamina development by the *GUN1*-dependent mechanism is beneficial for plant life in any respect when the plastid gene expression is impaired. The *GUN1*-dependent retrograde signal is considered to be the plastid-nucleus communication system to coordinate the nuclear gene expression with the changing plastid condition during chloroplast development [37], [39], [40]. Because plastid gene expression is affected differently by various biotic and abiotic stresses [64] and genetic mutations in nuclei and plastids, the coordinated regulation of nuclear genes is required for the successful development of photosynthetic apparatus from non-chloroplast plastids. However, when the plastid gene expression machinery is heavily impaired at the primordial stages, it is difficult to develop a fully functional photosynthetic apparatus. In such a case, full lamina expansion is risky because the cost of the lamina formation is not compensated for by little photosynthetic product. Therefore, the inhibition of lamina expansion by the *GUN1*-dependent mechanism can be interpreted as the avoidance of such wasteful development. From this viewpoint, it is suggestive that the *gun1* mutant becomes seedling lethal when transiently treated with lincomycin and in the *enf2* mutant background. This less viable *gun1* phenotype shows that the *GUN1*-dependent mechanism enables robust and sustainable plant development by optimizing the total photosynthetic efficiency even when plastid gene expression is impaired by internal and external fluctuations.

It is considered that the planar lamina morphology of seed plants had been evolved depending on the adaxial-abaxial asymmetry of gene expression in leaf primordia [90]. Meanwhile, the evolutionary advantage of the planar morphology is the efficient light reception in chloroplasts for photosynthesis, thus depends on ensuring the functional integrity of plastids. From this evolutionary point of view, the problem of how the regulatory system for adaxial-abaxial gene expression pattern and lamina expansion by plastid has been evolved is as important as that of how planar lamina morphology with adaxial-abaxial asymmetry has been evolved.

## Materials and Methods

### Plant Materials

*Arabidopsis thaliana* plants of Columbia (Col) accession with or without *FILpro:GFP 35Spro:miYFP-W* markers were used as the wild type. The *phb-1d/+* mutant (L.*er* accession background) was used after crossing to Col more than four times. The *enf2* mutants (*enf2-1 ene* and each single mutant) were isolated from the EMS-treated *FILpro:GFP* plants as described previously [29] and used for all analyses after backcrossing more than 4 times to parental line. *enf2-2* (SALK_063761) was obtained from the Arabidopsis Biological Resources Center (ABRC) at Ohio State University. The *flv* (CS3254 from ABRC), *gun1-1*, *gun2-1*, *gun3-1*, *gun4-1* and *gun5-1* mutants [75], [76] were previously described.

Plant seeds were sterilized and kept at 4°C in the dark for two days before sowing on 0.9% agar plates containing 1% sucrose and 0.5× Murashige-Skoog salt medium. For chemical treatments, each plate contained DEX (10 µM final concentration), lincomycin, erythromycin or norflurazon (see Results and Figures for the concentrations). For DEX treatment, growing seedlings were dipped in a DEX solution (50 µM) before being transferred to DEX plates. The plants were grown at 22°C and under continuous white fluorescent light of approximately 60 µmol photon m^−2^ s^−1^ except for the dark treatment, in which the plates were covered with aluminum foil. The *phb-1d/+* mutant was grown at 16°C to moderate its phenotypic severity. Some plants were transferred to or sown in soil when seeds were needed.

### Microscopy and Image Analysis

To observe GFP, YFP and VENUS marker expression patterns by confocal microscopy, plant samples were embedded into agarose gel, sectioned and observed as previously described [29]. The height of the observation plane from the leaf base was estimated from the section thickness, the section number from the meristem-containing section and the focal plane position within the observed section. The *FILpro:GFP*-positive areas in leaf primordia were measured using ImageJ v1.45s (National Institutes of Health, MD) as previously described [29]. In most of the observations, the plants were observed when the first leaf grew as big as the cotyledon.

To observe the VENUS expression area by stereoscopy, an SZX16 fluorescence stereoscope equipped with a GFP filter and a CCD camera DP72 (OLYMPUS, Japan) was used. From the acquired RGB color images, the green channel image was extracted, and the VENUS-positive areas in the adaxial epidermis and the adaxial-most mesophyll were measured using ImageJ. Normal stereoscopy images were acquired with the same stereoscope under white light illumination.

### Electron Microscopy

Scanning electron microscopy was performed as previously described [34]. The frozen leaves were cracked to observe the mesophyll structure.

For plastid ultrastructure observation, 8- or 16-day-old seedlings of the wild type and *enf2* were fixed by two steps in 0.05 M cacodylate buffer at pH 7.4. The first buffer contained 4% (w/v) paraformaldehyde and 1% (v/v) glutaraldehyde, and the second buffer contained 0.5% osmium tetraoxide. These fixation steps took overnight and two hours, respectively. Fixed samples were dehydrated with a series of ethanol solutions and transferred into propylene oxide. These processed samples were embedded into EPON 812 resin (TAAB Laboratories, UK). Ultrathin sections made with an Ultramicrotome (Leica, Austria) were stained by 4% uranyl acetate and 0.4% lead citrate. The ultrathin sections were examined with a transmission electron microscope H-7600 (Hitachi, Japan) at 80 kV.

### Plasmid Construction and Plant Transformation

All T-DNA transformation was performed by vacuum infiltration using the *Agrobacterium tumefaciens* strain ASE. Transgenic plants were screened for BASTA or Kanamycin resistance. The marker genes *FILpro:GFP* [15], *35Spro:miYFP-W* [29], *FILpro:CRE-GR* (see below) and *35Spro:loxP-Ter-loxP-VENUS* [42] were introduced into each mutant by genetic cross after T-DNA transformation into the wild type (Col). Double transgenic *FILpro:CRE-GR 35Spro:loxP-Ter-loxP-VENUS* plants were obtained by crossing the single transgenic lines and analyzed in the F1 generation.

For the *enf2* mutant complementation, a 6.1-kb *AT1G31410/ENF2* genomic fragment, including 2647 bp upstream from the start codon and 1199 bp downstream from the stop codon, was amplified from BAC#T8E3 (from ABRC) by PCR using the primers 5′-CGGGGTACCTGATTGAGAATGTGATGAAGG-5′ and 5′-GGCTCTAGAGACCTCGGGTAAAACCC-3′. This fragment was cloned into a modified pBluescript II SK+ (Agilent Technologies, CA), transferred to a binary vector, pGWB-NB1 [29], by the GATEWAY system (Life Technologies, CA), and then introduced into *enf2* plants. To express the VENUS-ENF2 fusion gene from the *ENF2* promoter, this 6.1-kb fragment was modified by insertion of VENUS CDS between the region of the putative plastid-transit peptide (predicted by TargetP, http://www.cbs.dtu.dk/services/TargetP/) and the PotD/F homology domain. For the *ene* complementation experiment, a 10.5-kb *AT1G80070/SUS2* genomic fragment, including 527 bp upstream from the start codon and 498 bp downstream from the stop codon, was amplified from the wild-type genome by PCR using the primers 5′-CGGGGTACCTGCCGATTCTCCCGGATTTTCA-5′ and 5′-ATGAGCTGCGGCCGCAGGAGGGATGATAAAACTGCTGT-3′. This 10.5-kb fragment was finally transferred into the vector pGWB-NB1 as the 6.1-kb *ENF2* fragment above. For the construction of *FILpro:CRE-GR* gene, the 6,011-bp *FIL* promoter [15] and the CRE-GR coding sequence in pML518 [42] were cloned in tandem into the multicloning site of a modified pBluescript II SK+ and finally transferred into the vector pGWB-NB1. The binary vector containing the *35Spro:loxP-Ter-loxP-VENUS* gene was previously described as pML988 [42].

### Positional Cloning

For mapping of the *ENF2* and *ENE* loci, the F2 population from the F1 hybrid between *enf2* (*enf2-1 ene*, Col accession background) and an L.*er* accession plant was used. The *ENF2* locus was mapped into a 54 kb region of chromosome I using polymorphism markers. Sequencing of all of the annotated genes (*AT1G31370* to *AT1G31540*) in this region found a mutation in the AT1G31410 gene. The *ENE* locus was linked to the lower arm terminus of chromosome I, and the mutation in the *AT1G80070/SUS2* gene was found by sequencing the linking region. New CAPS and SSLP markers were designed using information from the Monsanto Arabidopsis Polymorphism and L. *er* Sequence Collection (http://www.arabidopsis.org/Cereon/index.jsp).

### ENF2 mRNA Splicing Analysis

Total RNA was extracted from whole aerial parts of 8-day-old seedlings with the Plant RNeasy Mini Kit (QIAGEN, Germany). cDNA synthesis was performed using the SuperScriptIII First Strand Synthesis System for RT-PCR (Life Technologies) with a mixed primer of random hexanucleotide and Oligo(dT), and part of the ENF2 cDNA was amplified by PCR of 40 thermal cycles to saturate the amplification. The primer sequences were 5′-CCGATTGTCGTTACAGAGAATG-3′, 5′-AGGAGCTTTTTCTCCCGCATA-3′ and 5′-ACTCGTCCTCCTCTTTGTTC-3′ (blue, pink and orange arrows in Figure 5A, respectively). The PCR products were analyzed by conventional agarose gel-electrophoresis, and each fragment of a distinct size was sequenced to identify the abnormal exon junctions. The same PCR products were also applied to microfluidics-based electrophoresis, using a 2100 Bioanalyzer (Agilent Technologies, CA), to detect the normally spliced mRNAs.

### qRT-PCR Analysis for the Plastid Genes

Total RNA was extracted from a shoot apex sample containing the apical meristem and only approximately the seven youngest leaves of not more than 500 µm in height. The cotyledons and hypocotyl were eliminated as much as possible. To compare the wild type, *enf2*, norflurazone-treated plant and *enf2 gun1* at comparable stages with similar leaf numbers and sizes, their shoot apices were collected at 5.5, 7, 10 and 13 days old, respectively. cDNA synthesis was performed with QuantiTect Reverse Transcription Kit (QIAGEN) accordingly to the manufacturer's instructions. For quantitative PCR, QuantiTect SYBR Green PCR Kit (QIAGEN) and the primer sets shown in Table S1 were used. The data collection and analysis were performed with Rotor-Gene Q (QIAGEN) and the Rotor-Gene 6000 series software 1.7 (QIAGEN). Some primer sequences were based on the previous study [66]. The average expression levels and the standard errors were calculated from biological quadruplicate data.

### Mathematical Model and Simulations

All calculations and related graphical representation were performed with Mathematica 7.0 (Wolfram Research, IL). The Hill coefficients for the functions *f_i_* and *g_i_* were set to n = 2 and n = 1, respectively, because the transcriptional repression is generally implemented by dimerized or larger complexes of transcription factors and mRNA cleavage by small RNA is a one-to-one reaction, but other higher values of the Hill coefficients gave similar results (data not shown). For numerical simulations, equations (1–2) were discretized in time with the time step Δt = 0.2 by the fourth-order Runge–Kutta method, and the reflective boundary condition was imposed.

### Accession Numbers

Sequence data from this article can be found in GenBank/EMBL databases under the following accession numbers: AtENF2, NP_174426.2 (*Arabidopsis thaliana*); NtENF2, XP_003628983.1 (*Medicago truncatula*); OsENF2, CAE01823.2 (*Oryza sativa*); ZmENF2, NP_001146059.1 (*Zea mays*); SmENF2, XP_002991704.1 (*Selaginella moellendorffii*); PpENF2, XP_001760514.1 (*Physcomitrella patens*); CsENF2, EIE23974.1 (*Coccomyxa subellipsoidea*); VcENF2, XP_002946447.1 (*Volvox carteri*); AvENF2, YP_323566.1 (*Anabaena variabilis*); NsENF2, ZP_01631537.1 (*Nodularia spumigena*); NaENF2, YP_003722186.1 (*Nostoc azollae*); BbPotD, AAB91528.1 (*Borrelia burgdorferi*); MhLpp38, AAA84748.1 (*Mannheimia haemolytica*); PfPotD, AAC15511.1 (*Pseudomonas fluorescens*); AaPotD, AAC27498.1 (*Aggregatibacter actinomycetemcomitans*); TpPotD, AAC65630.1 (*Treponema pallidum*); HiPot2, P44731.2 (*Haemophilus influenzae*); HiPot1, P45168.1 (*Haemophilus influenzae*); EcPotF, AAC73941.1 (*Escherichia coli*) and EcPotD, NP_415641.1 (*Escherichia coli*).

## Supporting Information

Figure S1 A simple mathematical model generates the shifting boundary between gene expression domains. (A) The function *f_j_*(z) is a decreasing function ranged between *p_j_* and *p_j_* + *r_j_*. (B) The function *g_j_*(z) is an increasing function ranged between *d_j_* and *d_j_* + *c_j_*. (C–F) The relationships between the parameter and the boundary dynamics type. *p_1_* and *p_2_* (C), *r_1_* and *r_2_* (D), *d_1_* and *d_2_* (E), *c_1_* and *c_2_* (F), are varied from the parameter set for Figure 1C.
(TIF)

Figure S2 The expression patterns of *FILpro:GFP* and *35Spro:miYFP-W* in leaf primordia at various developmental stages. (A, B) The boxed region in Figure 1B. *35Spro:miYFP-W* signal (A) and *FILpro:GFP* signal (B) are individually shown with schematic illustrations below. (C–P) The leaf primordia at around P0 stage (C, D), P1 stage (20-µm-long) (E, F), around P6 stage (G–J) and around P10 stage (1,300-µm-long) (K–P). Confocal images show *FILpro:GFP* (green) and *35Spro:miYFP-W* (magenta) signals in longitudinal (C) and transverse (D–F, L–P) sections and the section planes parallel to the lamina (H–J). (H–J), A Z-series of optical sections with 5 µm intervals from the same primordium. (L–P), A series of transverse sections from the same primordium. The approximate heights of the observation planes from the leaf base are indicated. (G, K), The scanning electron microscope images showing the elongation of marginal tip cells at each stage. Arrowheads indicate the marginal tip cells elongating (white) and not elongated yet (black). Scale bars represent 50 µm (C), 20 µm (D–F), 100 µm (G–J, L–P) and 500 µm (K).
(TIF)

Figure S3 The efficiency and spatio-temporal specificity of the CRE/loxP recombination. (A, B) Confocal images showing the fluorescent signals from VENUS (yellow) of *FILpro:CRE-GR 35Spro:loxP-Ter-loxP-VENUS* system (A) and *FILpro:GFP* (green) and *35Spro:miYFP-W* (magenta) (B) in a longitudinal section of a reproductive shoot apex. Arrowheads, the center of the flower primordium; “+”, the center of the shoot apical meristem; Scale bars, 50 µm. (C–F) Stereoscopic fluorescent images of the third leaves from the *FILpro:CRE-GR 35Spro:loxP-Ter-loxP-VENUS* plants grown on DEX containing (C, D) and DEX-free (E, F) medium. ad, adaxial view; ab, abaxial view; Scale bars, 1 mm. The average sizes (%) and the standard errors of the epidermal VENUS expression areas measured from more than ten of such images are shown below each image. (G, H) Confocal images showing the VENUS (yellow) expression pattern in the transverse sections of *FILpro:CRE-GR 35Spro:loxP-Ter-loxP-VENUS* plants at 6 hours (A) and 12 hours (B) after DEX application. Each differential interference contrast (DIC) image is shown on the right. “+”, the center of the shoot apical meristem; ad, adaxial side; ab, abaxial side; Scale bars, 50 µm.
(TIF)

Figure S4 The VENUS expression patterns in the *FILpro:CRE-GR 35Spro:loxP-Ter-loxP-VENUS* leaves. (A–C) Fluorescent stereoscopic images of the third leaves treated with DEX from the day 4 (A), day 6 (B) and day 8 (C). left panel, both fluorescence of VENUS (yellow-green) and chlorophyll (red); right, VENUS fluorescence alone. (D–H) Confocal images showing VENUS fluorescence (yellow) and chlorophyll fluorescence (red) in the leaf sections. The corresponding section planes are indicated in (A–C) as broken lines. Scale bars represent 1 mm (A–C), 500 µm (D–H).
(TIF)

Figure S5 The *enf2* phenotypes. (A–D) Confocal images of transverse sections showing *FILpro:GFP* (green) and *35Spro:miYFP-W* (magenta) marker expression in the wild type (A, B) and *enf2* (C, D) leaf primordia. *FIL*-expression area sizes (%) are indicated at the bottom right. The specimens in (A, C) are at around the P2 stage, and those in (B, D) are at around the P4 stage of leaf development. (E) The VENUS expression area sizes (%, y-axis) in wild-type (black lines) and *enf2* (red lines) leaves harboring *FILpro:CRE-GR 35Spro:loxP-Ter-loxP-VENUS*. The x-axis represents the DEX treatment dates. The data for the adaxial epidermis and the adaxial-most mesophyll are shown as broken lines and normal lines, respectively. Bars indicate the standard errors. (F–N) Stereoscopic images showing VENUS fluorescence (yellow-green) of *FILpro:CRE-GR 35Spro:loxP-Ter-loxP-VENUS* in the third leaves of 15-day-old *enf2*. The DEX treatment dates are indicated at the bottom left. (F–M) are adaxial-side views, and (N) is an abaxial-side view. (O) The needle-like structure of an *enf2* leaf. n.s., not significantly different (*p*≥0.05, t-test) between the wild type and *enf2*. Scale bars represent 50 µm (A–D) and 1 mm (F–O).
(TIF)

Figure S6 The transgene *ENF2* and *ENF2pro:VENUS-ENF2* rescue the *enf2* phenotype. The stereoscopic images of seedlings (left), confocal images showing *FILpro:GFP* expression patterns (middle) and the corresponding DIC images (right) are shown for each plant of the wild type (A), *enf2* (B) and transformed *enf2* (C, D). The transgenes *ENF2* (C) and *ENF2pro:VENUS-ENF2* (D) are introduced into *enf2*. Scale bars represent 2 mm (left panels) and 100 µm (middle and right panels).
(TIF)

Figure S7 The effects of the *ene* mutation. (A) The electropherogram of the *ENF2* RT-PCR products (Figure 5B right panel) from the wild type, *enf2-1* and *enf2-1 ene* (*enf2*) plants. Each line shows average of each triplicate data. The peaks of normally spliced *ENF2* mRNA are indicated by red arrowheads and displayed in a close-up view (inset). (B–F) Seedlings of *enf2-2*, the wild type, *enf2-1*, *ene* and *enf2-1 ene* (*enf2*). (G) *FIL*-expression area sizes (%, y-axis) at different stages (grouped by section area sizes, x-axis) of wild-type, *enf2-1*, *ene* and *enf2-1 ene* (*enf2*) leaf primordia. Bars indicate the standard errors. n.s., not significantly different; *, significantly different (*p*<0.05, t-test) between the wild type and each mutant. (H) Schematic representation of the *AT1G80070/SUS2/ENE* gene. (I–L) Seedlings of the wild type (I), *enf2-1* single mutant (J), *enf2* mutant (K) and *SUS2*-transformed *enf2* (L). These plants were grown under 16°C to emphasize the growth difference between *enf2-1* single mutant and *enf2* mutant. Scale bars represent 1 mm (B–F) and 5 mm (I–L).
(TIF)

Figure S8 Amino acid sequence alignment of ENF2 and PotD homologs. Only the part mainly interacting with polyamine in PotD is shown. Red represents the residues indispensable for the interaction with polyamine in PotD and conserved in other homologs [55]. Green represents the ENF2 residues corresponding to the red parts and conserved among other homologs.
(TIF)

Figure S9 The plastid in *enf2* shows defects in chloroplast development and plastid gene expression profile. (A–F) The transmission electron microscope images showing the plastid in the wild type (A–C) and in *enf2* (D–F). All images indicate subepidermal cells. The stages are meristem (A, D), leaf primordia at the P4–P6 stage (B, E) and mature leaves (C, F). Scale bars represent 1 µm (A, B, D, E), 2 µm (C, F). (G) qRT-PCR results showing the transcript abundance of plastid genes encoding proteins (left eighty) and 16S and 23S rRNA (right two) in *enf2*. The results were normalized to 18S rRNA, and the *enf2* values relative to the wild type are represented as log2 values. The protein-encoding genes are sorted on the x-axis by their location on the plastid genome, which corresponds to the order in Table S1. Error bars indicate standard errors.
(TIF)

Figure S10 The effects of norflurazon treatment and dark growth. (A–C) Seedlings of the norflurazon-treated plant (A) and the dark-grown plant (B, C). (B), A close-up view of a leaf lamina of dark-grown seedling. (D, E) Confocal images of transverse sections showing *FILpro:GFP* (green) and *35Spro:miYFP-W* (magenta) marker expression in each plant of (A, B). (F) qRT-PCR results showing the transcript abundance of plastid genes encoding proteins (left eleven) and 16S and 23S rRNA (right two) in norflurazon-treated shoot apex (grey). The results were normalized to 18S rRNA, and the relative values to the untreated plants are represented as log2 values. Error bars indicate standard errors. The data of *enf2* mutant (white) are the same as in Figure S9. (G) A wild-type seedling treated with 25 µM norflurazon and 450 µM lincomycin. (H) An *enf2* seedling treated with 150 µM lincomycin. (I) A wild-type seedling treated with 450 µM lincomycin. Scale bars represent 1 mm (A–C, G–I) and 50 µm (D, E).
(TIF)

Figure S11 The involvement of plastid retrograde signal in the developmental effects from the plastid gene expression. (A) A gun5 seedling treated with 230 µM lincomycin (B) An *enf2 gun5* seedling. (C) *FIL*-expression area sizes (%, y-axis) at different stages (grouped by section area sizes, x-axis) of the wild-type and *gun1* leaf primordia. Bars indicate the standard errors. n.s., not significantly different (*p*≥0.05, t-test) between the wild type and *gun1*. (D) The qRT-PCR results showing the transcript abundance of plastid genes encoding proteins (left eleven) and 16S and 23S rRNA (right two) in *enf2 gun1* (grey) shoot apex. The results were normalized to 18S rRNA, and the relative mutant values to the wild type are represented as log2 values. Error bars indicate standard errors. The data of *enf2* mutant (white) are the same as in Figure S9. (E, F) The wild type and *gun1* seedlings grown on the standard medium after growth on the lincomycin-containing medium for two weeks each. Scale bars represent 1 mm.
(TIF)

Figure S12 The model of mutual repression and mobility is applicable to simulation of *phb-1d/+* phenotype. Computer simulation results with an assumed wild type parameter set (A): *p_1_* = *p_2_* = 0.1, *r_1_* = 2.0, *r_2_* = 1.8, *d_1_* = *d_2_* = 0.1, *c_1_* = *c_2_* = 2.0 and *D_AD_* = *D_AB_* = 0.1, and an assumed *phb-1d/+* parameter set (B): *p_1_* = *p_2_* = 0.1, *r_1_* = 2.0, *r_2_* = 1.8, *d_1_* = *d_2_* = 0.1, *c_1_ = *1.9, *c_2_* = 2.0 and *D_AD_* = *D_AB_* = 0.1. Note that *c_1_* value is smaller in B than in A. The adaxial-most cell was fixed in the *AD-*expressing state through each simulation, and the other abaxial five cells were set to be in the *AB-*expressing state as their initial conditions and followed the equations (1–4) during the simulations.
(TIF)

Table S1 Primers used for plastid gene expression analyses.
(XLS)

Text S1 The efficiency and spatio-temporal specificity of the CRE/loxP recombination.
(PDF)
