# Supplementary figures and images for: Pattern Dynamics in Adaxial-Abaxial Specific Gene Expression Are Modulated by a Plastid Retrograde Signal during Arabidopsis thaliana Leaf Development
Source: PLoS Genet. 2013 Jul 25;9(7):e1003655. doi: 10.1371/journal.pgen.1003655 (PMC3723520; doi:10.1371/journal.pgen.1003655)

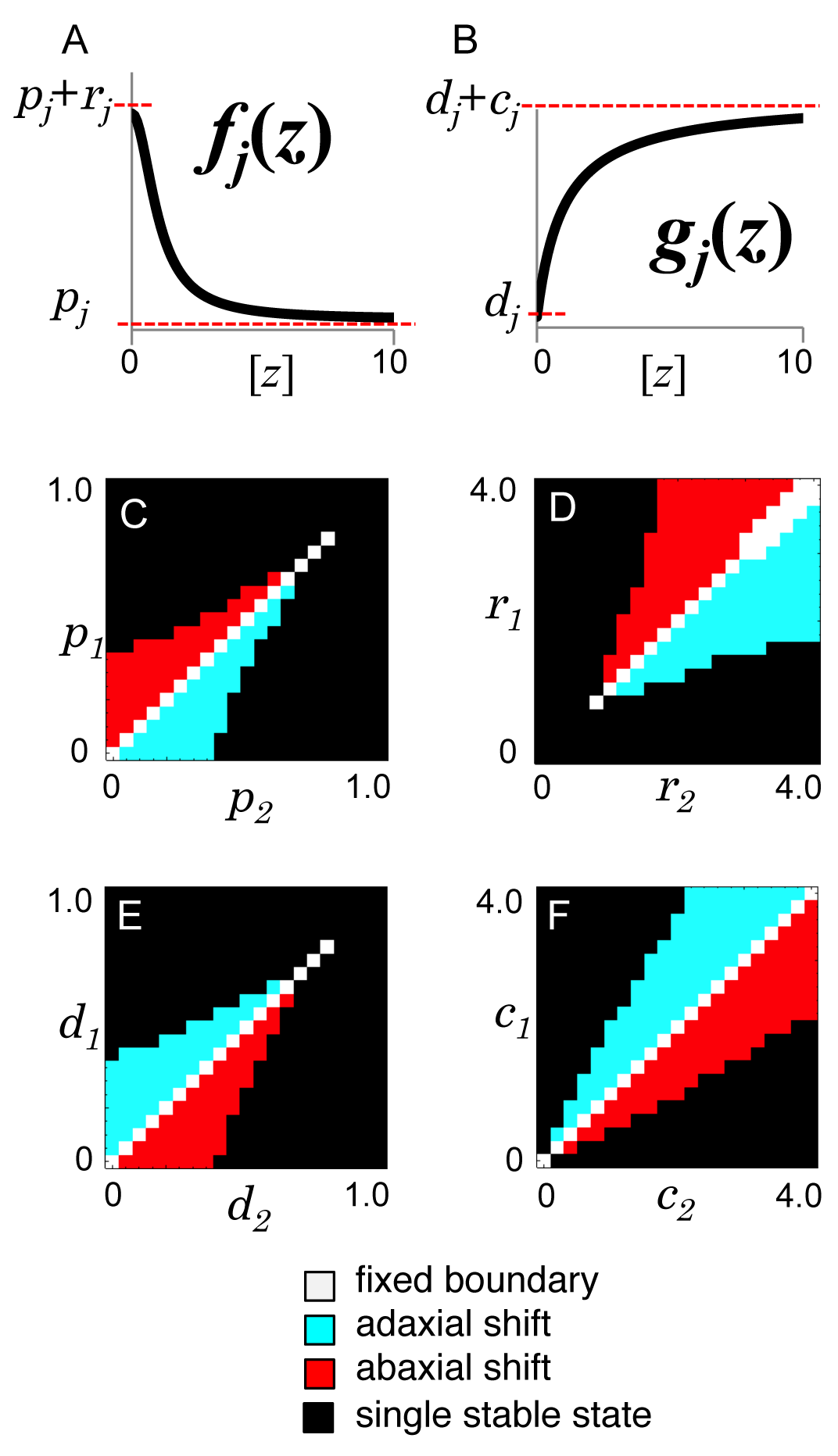

Supplement: Figure S1 — A simple mathematical model generates the shifting boundary between gene expression domains. (A) The function fj(z) is a decreasing function ranged between pj and pj + rj. (B) The function gj(z) is an increasing function ranged between dj and dj + cj. (C–F) The relationships between the parameter and the boundary dynamics type. p1 and p2 (C), r1 and r2 (D), d1 and d2 (E), c1 and c2 (F), are varied from the parameter set for Figure 1C. (TIF) [file pgen.1003655.s001.tif]

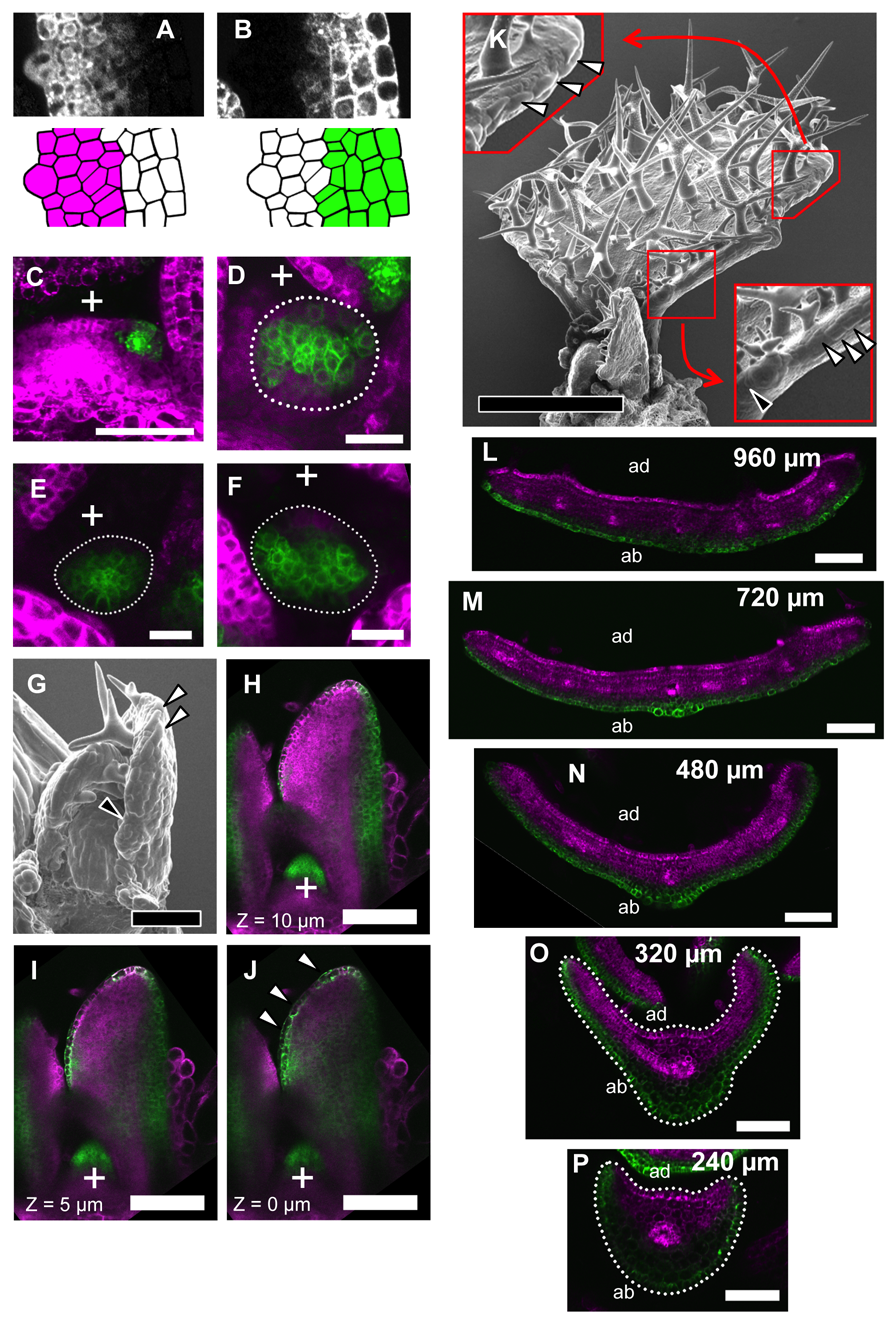

Supplement: Figure S2 — The expression patterns of FILpro:GFP and 35Spro:miYFP-W in leaf primordia at various developmental stages. (A, B) The boxed region in Figure 1B. 35Spro:miYFP-W signal (A) and FILpro:GFP signal (B) are individually shown with schematic illustrations below. (C–P) The leaf primordia at around P0 stage (C, D), P1 stage (20-µm-long) (E, F), around P6 stage (G–J) and around P10 stage (1,300-µm-long) (K–P). Confocal images show FILpro:GFP (green) and 35Spro:miYFP-W (magenta) signals in longitudinal (C) and transverse (D–F, L–P) sections and the section planes parallel to the lamina (H–J). (H–J), A Z-series of optical sections with 5 µm intervals from the same primordium. (L–P), A series of transverse sections from the same primordium. The approximate heights of the observation planes from the leaf base are indicated. (G, K), The scanning electron microscope images showing the elongation of marginal tip cells at each stage. Arrowheads indicate the marginal tip cells elongating (white) and not elongated yet (black). Scale bars represent 50 µm (C), 20 µm (D–F), 100 µm (G–J, L–P) and 500 µm (K). (TIF) [file pgen.1003655.s002.tif]

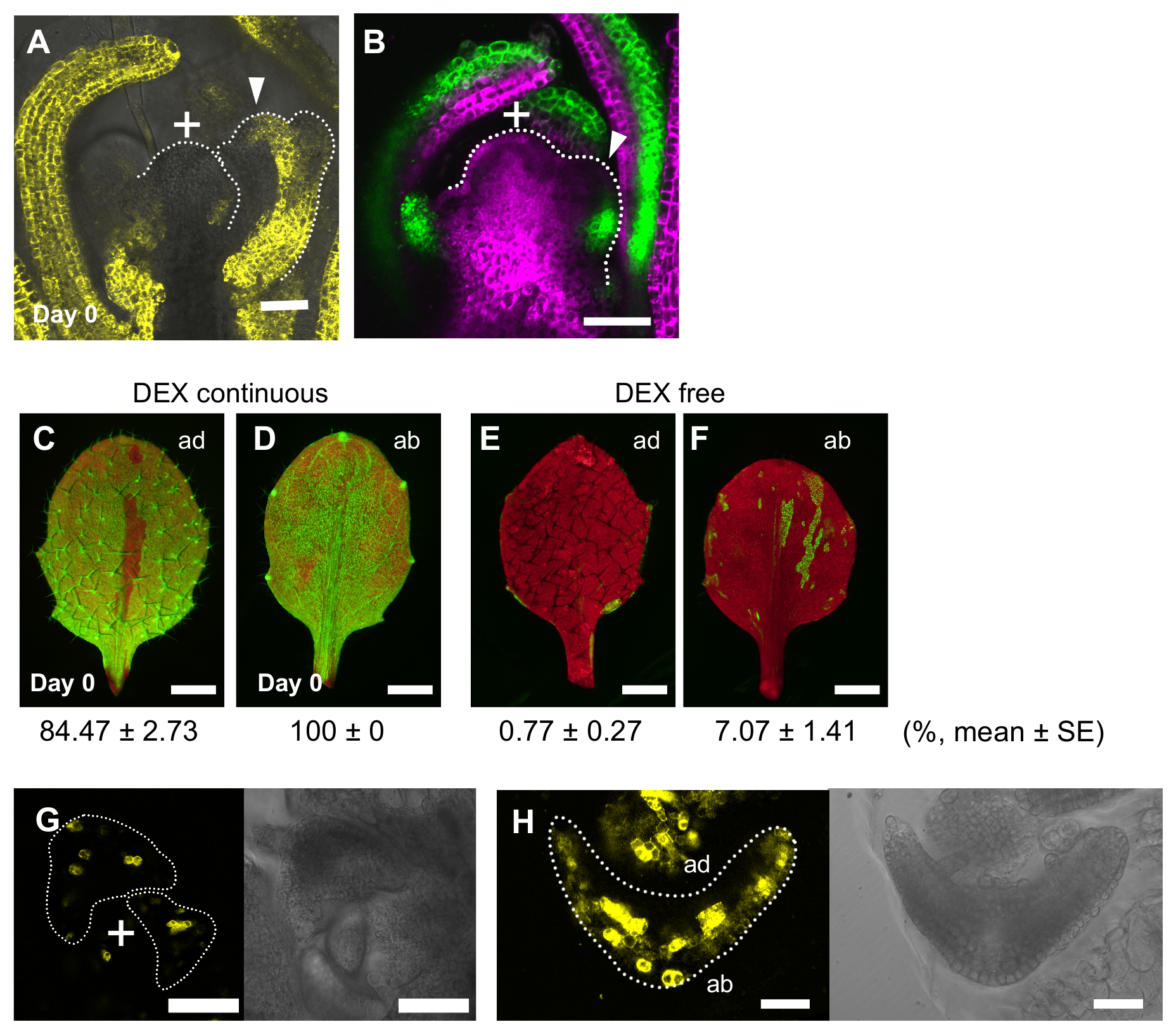

Supplement: Figure S3 — The efficiency and spatio-temporal specificity of the CRE/loxP recombination. (A, B) Confocal images showing the fluorescent signals from VENUS (yellow) of FILpro:CRE-GR 35Spro:loxP-Ter-loxP-VENUS system (A) and FILpro:GFP (green) and 35Spro:miYFP-W (magenta) (B) in a longitudinal section of a reproductive shoot apex. Arrowheads, the center of the flower primordium; “+”, the center of the shoot apical meristem; Scale bars, 50 µm. (C–F) Stereoscopic fluorescent images of the third leaves from the FILpro:CRE-GR 35Spro:loxP-Ter-loxP-VENUS plants grown on DEX containing (C, D) and DEX-free (E, F) medium. ad, adaxial view; ab, abaxial view; Scale bars, 1 mm. The average sizes (%) and the standard errors of the epidermal VENUS expression areas measured from more than ten of such images are shown below each image. (G, H) Confocal images showing the VENUS (yellow) expression pattern in the transverse sections of FILpro:CRE-GR 35Spro:loxP-Ter-loxP-VENUS plants at 6 hours (A) and 12 hours (B) after DEX application. Each differential interference contrast (DIC) image is shown on the right. “+”, the center of the shoot apical meristem; ad, adaxial side; ab, abaxial side; Scale bars, 50 µm. (TIF) [file pgen.1003655.s003.tif]

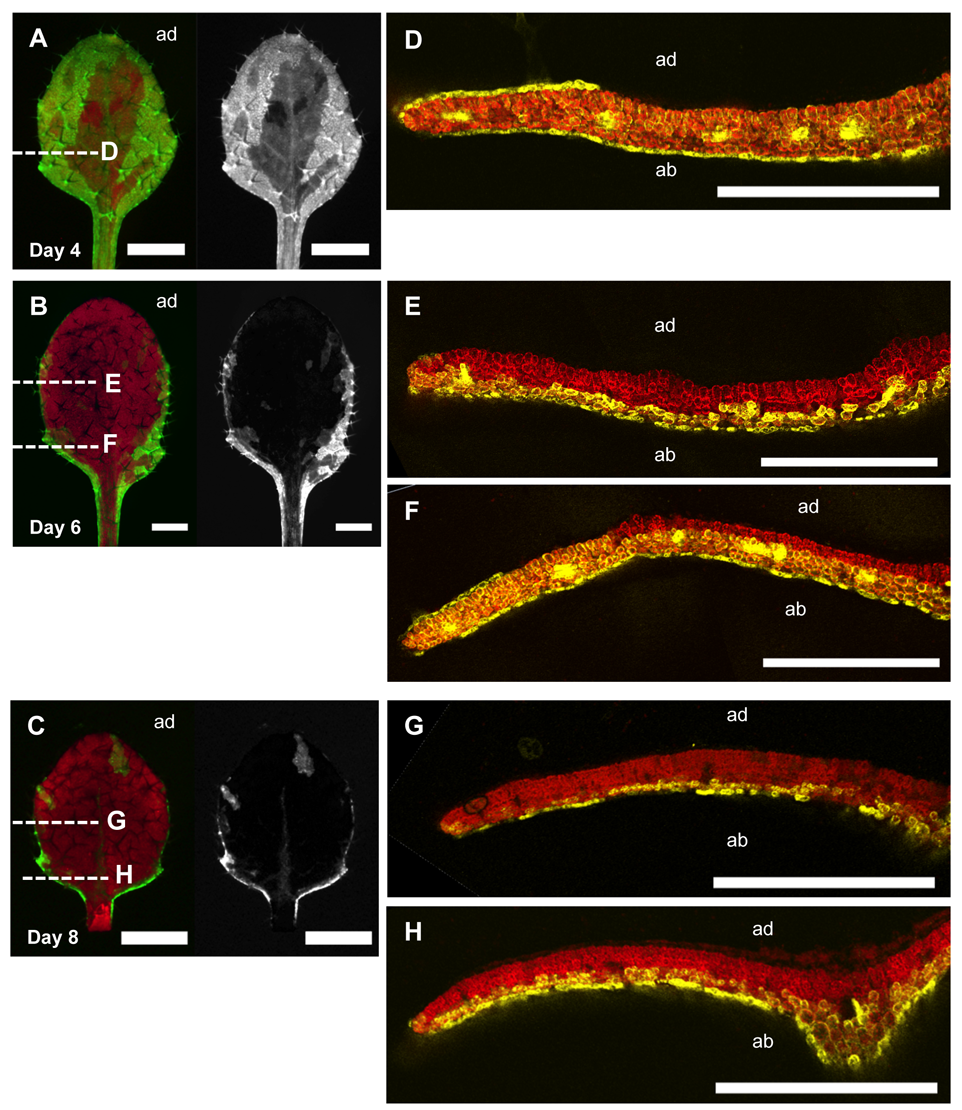

Supplement: Figure S4 — The VENUS expression patterns in the FILpro:CRE-GR 35Spro:loxP-Ter-loxP-VENUS leaves. (A–C) Fluorescent stereoscopic images of the third leaves treated with DEX from the day 4 (A), day 6 (B) and day 8 (C). left panel, both fluorescence of VENUS (yellow-green) and chlorophyll (red); right, VENUS fluorescence alone. (D–H) Confocal images showing VENUS fluorescence (yellow) and chlorophyll fluorescence (red) in the leaf sections. The corresponding section planes are indicated in (A–C) as broken lines. Scale bars represent 1 mm (A–C), 500 µm (D–H). (TIF) [file pgen.1003655.s004.tif]

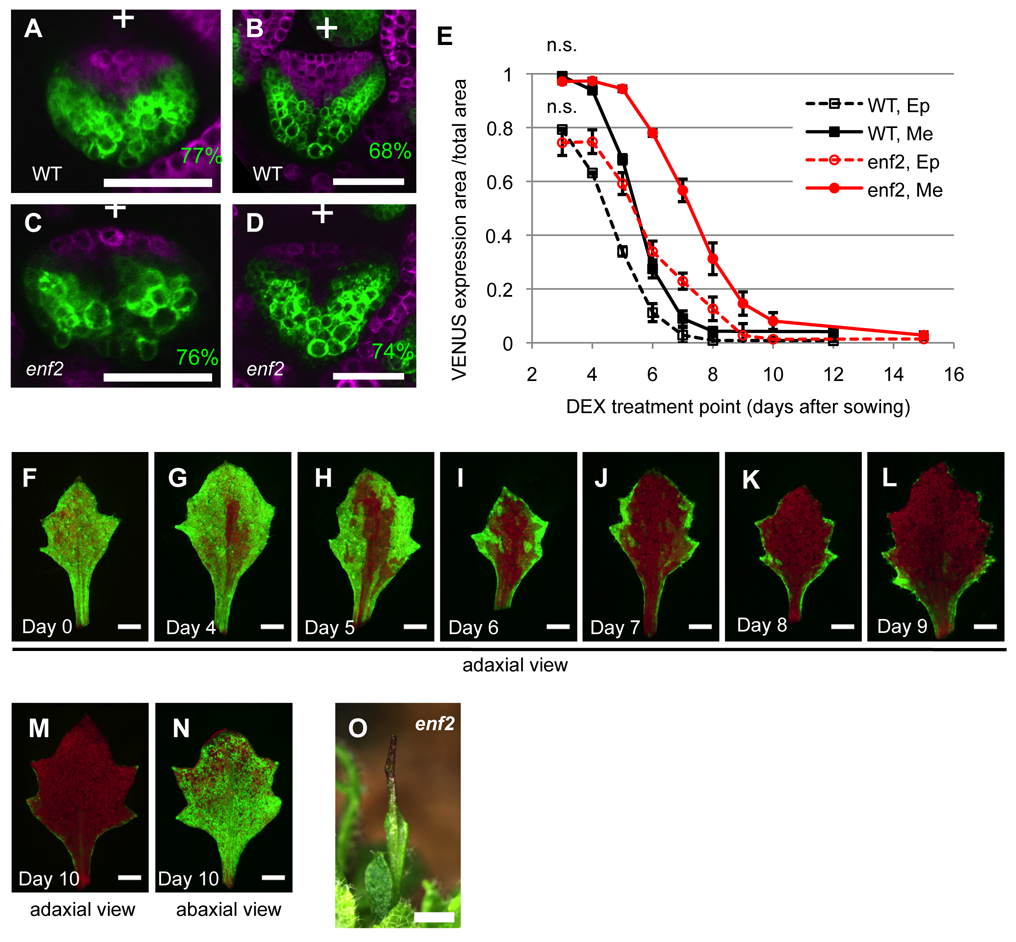

Supplement: Figure S5 — The enf2 phenotypes. (A–D) Confocal images of transverse sections showing FILpro:GFP (green) and 35Spro:miYFP-W (magenta) marker expression in the wild type (A, B) and enf2 (C, D) leaf primordia. FIL-expression area sizes (%) are indicated at the bottom right. The specimens in (A, C) are at around the P2 stage, and those in (B, D) are at around the P4 stage of leaf development. (E) The VENUS expression area sizes (%, y-axis) in wild-type (black lines) and enf2 (red lines) leaves harboring FILpro:CRE-GR 35Spro:loxP-Ter-loxP-VENUS. The x-axis represents the DEX treatment dates. The data for the adaxial epidermis and the adaxial-most mesophyll are shown as broken lines and normal lines, respectively. Bars indicate the standard errors. (F–N) Stereoscopic images showing VENUS fluorescence (yellow-green) of FILpro:CRE-GR 35Spro:loxP-Ter-loxP-VENUS in the third leaves of 15-day-old enf2. The DEX treatment dates are indicated at the bottom left. (F–M) are adaxial-side views, and (N) is an abaxial-side view. (O) The needle-like structure of an enf2 leaf. n.s., not significantly different (p≥0.05, t-test) between the wild type and enf2. Scale bars represent 50 µm (A–D) and 1 mm (F–O). (TIF) [file pgen.1003655.s005.tif]

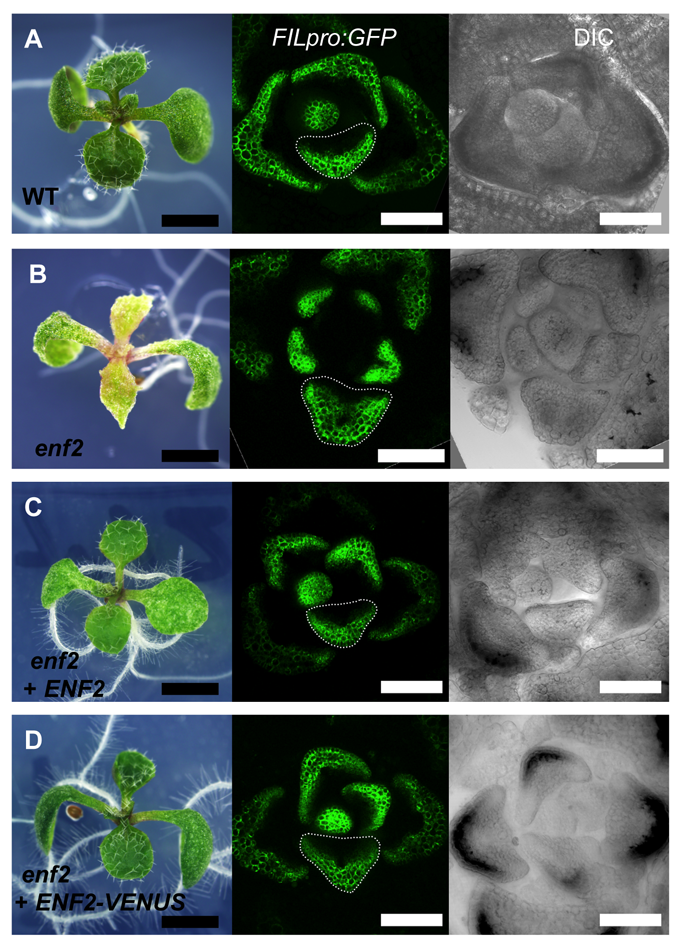

Supplement: Figure S6 — The transgene ENF2 and ENF2pro:VENUS-ENF2 rescue the enf2 phenotype. The stereoscopic images of seedlings (left), confocal images showing FILpro:GFP expression patterns (middle) and the corresponding DIC images (right) are shown for each plant of the wild type (A), enf2 (B) and transformed enf2 (C, D). The transgenes ENF2 (C) and ENF2pro:VENUS-ENF2 (D) are introduced into enf2. Scale bars represent 2 mm (left panels) and 100 µm (middle and right panels). (TIF) [file pgen.1003655.s006.tif]

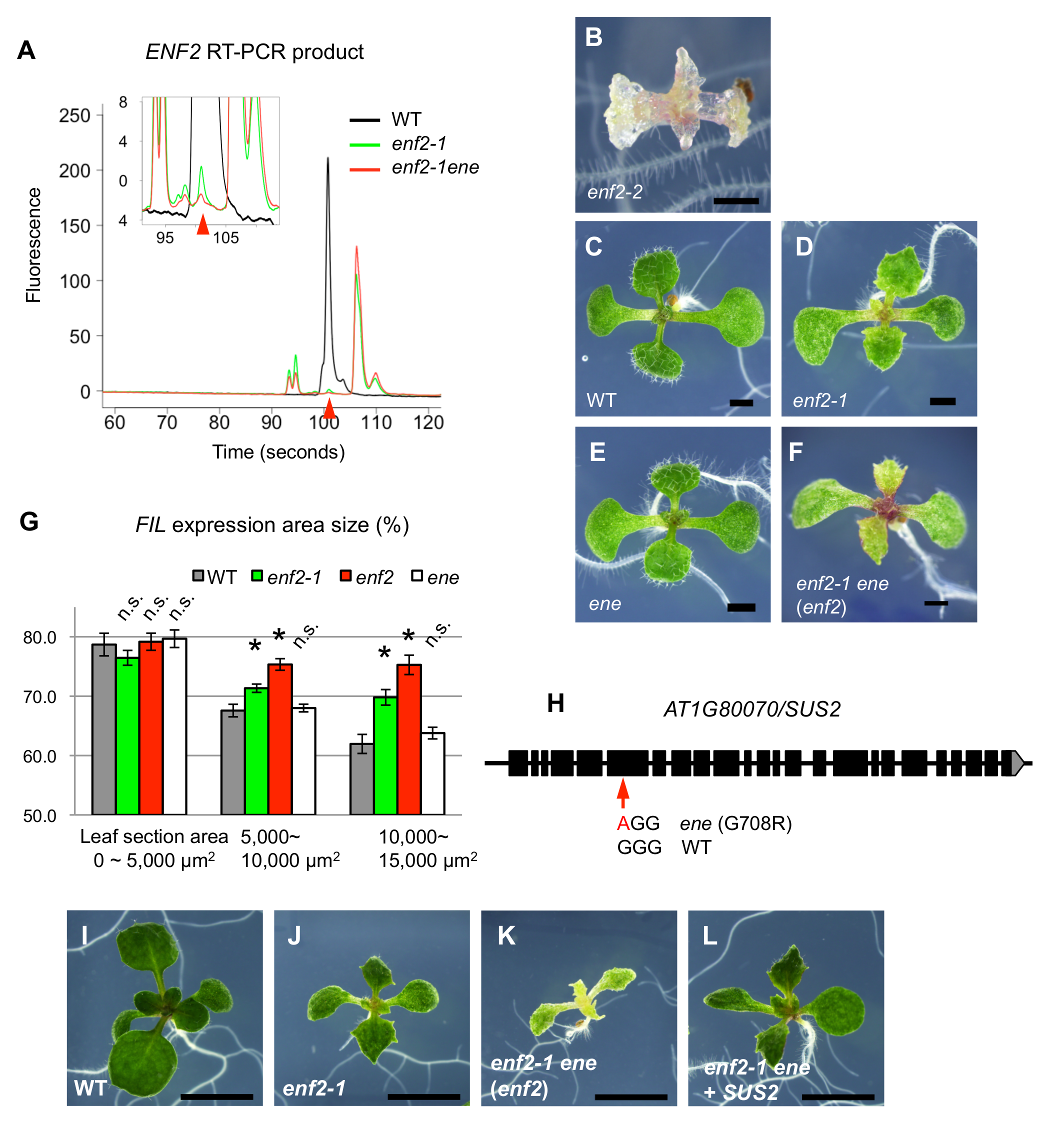

Supplement: Figure S7 — The effects of the ene mutation. (A) The electropherogram of the ENF2 RT-PCR products (Figure 5B right panel) from the wild type, enf2-1 and enf2-1 ene (enf2) plants. Each line shows average of each triplicate data. The peaks of normally spliced ENF2 mRNA are indicated by red arrowheads and displayed in a close-up view (inset). (B–F) Seedlings of enf2-2, the wild type, enf2-1, ene and enf2-1 ene (enf2). (G) FIL-expression area sizes (%, y-axis) at different stages (grouped by section area sizes, x-axis) of wild-type, enf2-1, ene and enf2-1 ene (enf2) leaf primordia. Bars indicate the standard errors. n.s., not significantly different; *, significantly different (p<0.05, t-test) between the wild type and each mutant. (H) Schematic representation of the AT1G80070/SUS2/ENE gene. (I–L) Seedlings of the wild type (I), enf2-1 single mutant (J), enf2 mutant (K) and SUS2-transformed enf2 (L). These plants were grown under 16°C to emphasize the growth difference between enf2-1 single mutant and enf2 mutant. Scale bars represent 1 mm (B–F) and 5 mm (I–L). (TIF) [file pgen.1003655.s007.tif]

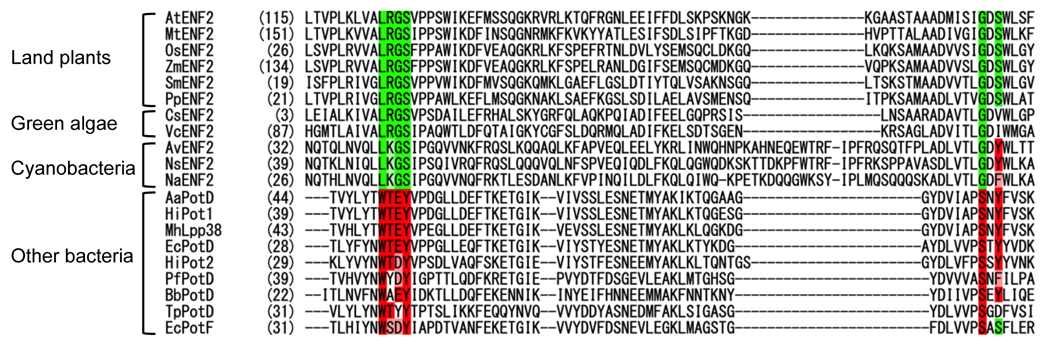

Supplement: Figure S8 — Amino acid sequence alignment of ENF2 and PotD homologs. Only the part mainly interacting with polyamine in PotD is shown. Red represents the residues indispensable for the interaction with polyamine in PotD and conserved in other homologs [55]. Green represents the ENF2 residues corresponding to the red parts and conserved among other homologs. (TIF) [file pgen.1003655.s008.tif]

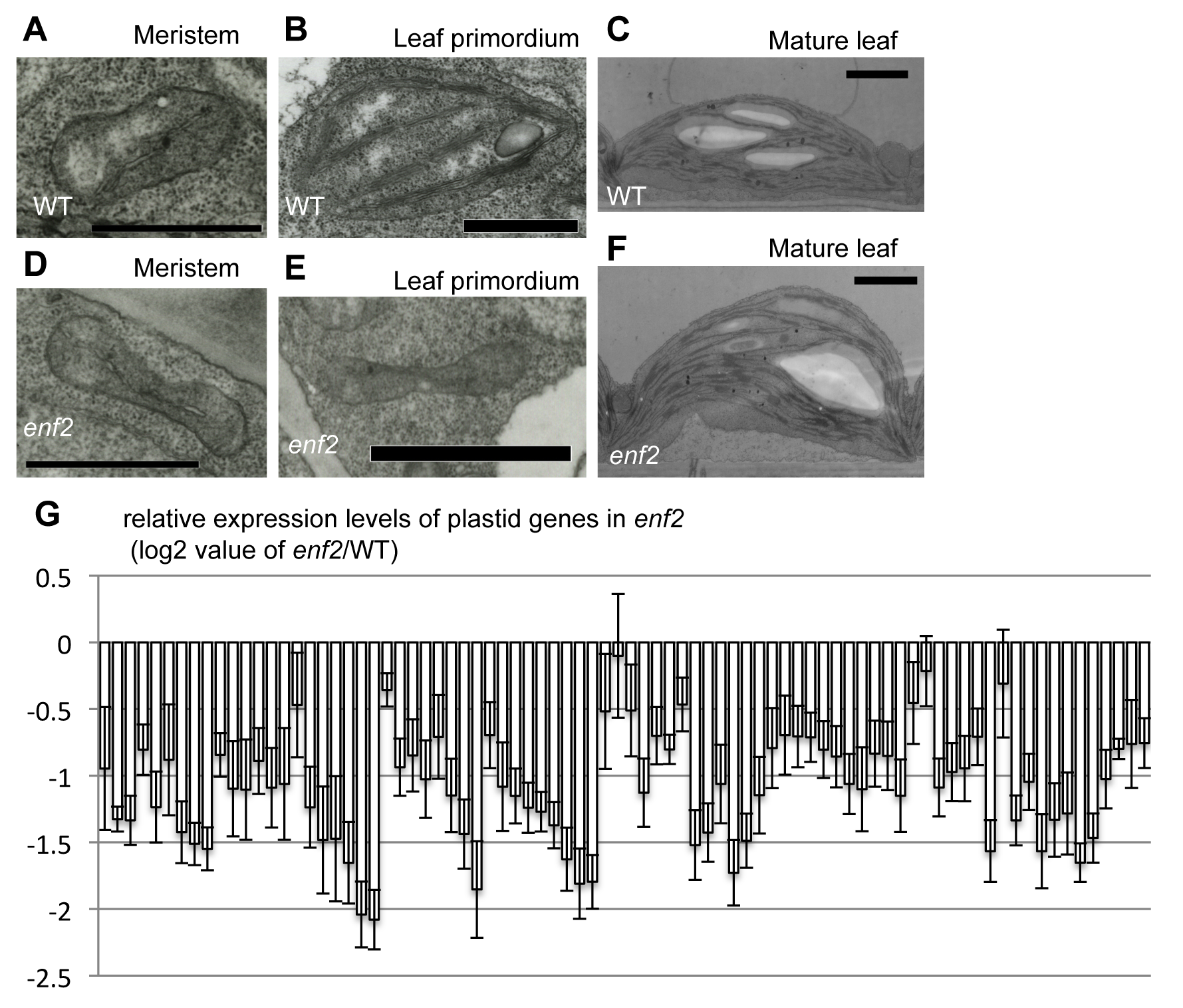

Supplement: Figure S9 — The plastid in enf2 shows defects in chloroplast development and plastid gene expression profile. (A–F) The transmission electron microscope images showing the plastid in the wild type (A–C) and in enf2 (D–F). All images indicate subepidermal cells. The stages are meristem (A, D), leaf primordia at the P4–P6 stage (B, E) and mature leaves (C, F). Scale bars represent 1 µm (A, B, D, E), 2 µm (C, F). (G) qRT-PCR results showing the transcript abundance of plastid genes encoding proteins (left eighty) and 16S and 23S rRNA (right two) in enf2. The results were normalized to 18S rRNA, and the enf2 values relative to the wild type are represented as log2 values. The protein-encoding genes are sorted on the x-axis by their location on the plastid genome, which corresponds to the order in Table S1. Error bars indicate standard errors. (TIF) [file pgen.1003655.s009.tif]

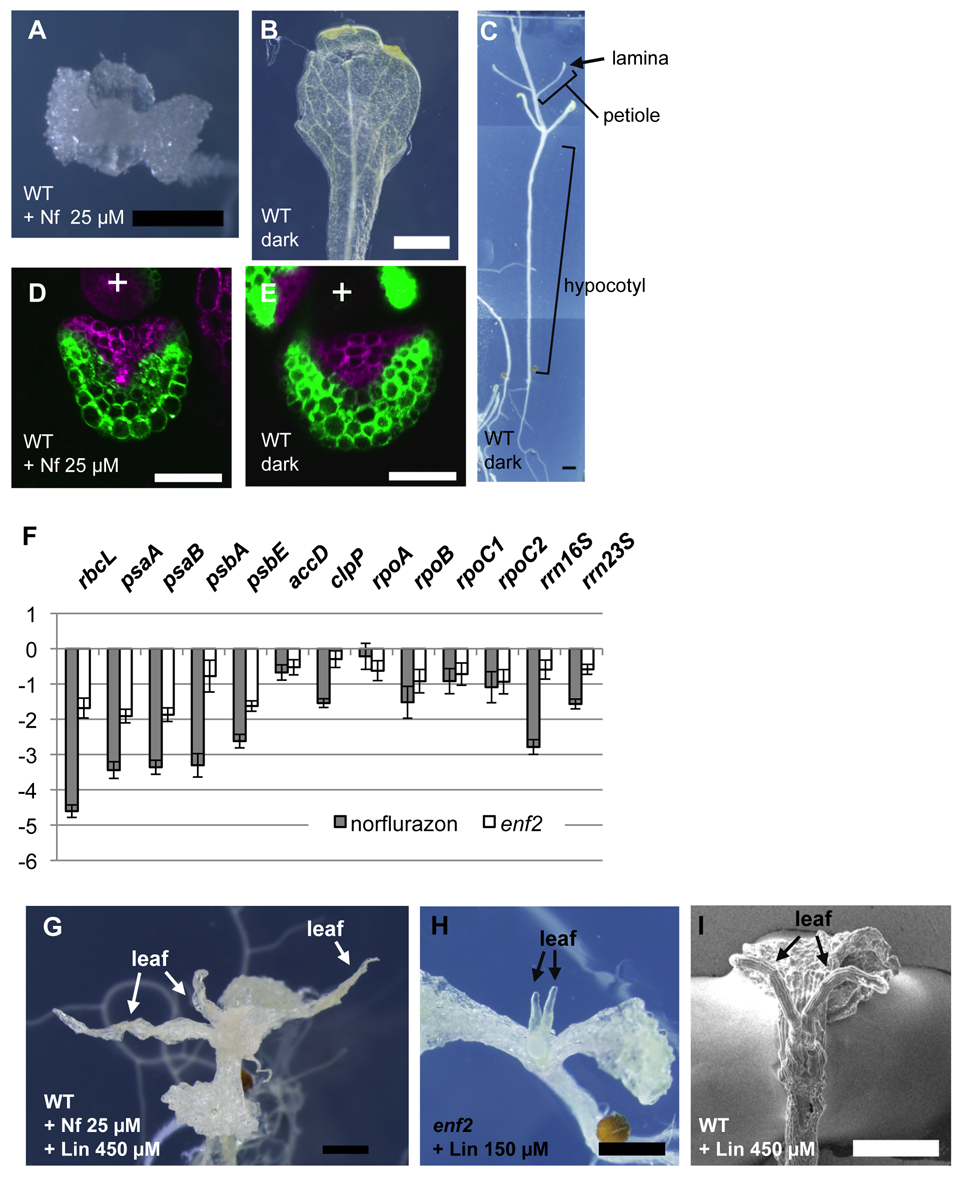

Supplement: Figure S10 — The effects of norflurazon treatment and dark growth. (A–C) Seedlings of the norflurazon-treated plant (A) and the dark-grown plant (B, C). (B), A close-up view of a leaf lamina of dark-grown seedling. (D, E) Confocal images of transverse sections showing FILpro:GFP (green) and 35Spro:miYFP-W (magenta) marker expression in each plant of (A, B). (F) qRT-PCR results showing the transcript abundance of plastid genes encoding proteins (left eleven) and 16S and 23S rRNA (right two) in norflurazon-treated shoot apex (grey). The results were normalized to 18S rRNA, and the relative values to the untreated plants are represented as log2 values. Error bars indicate standard errors. The data of enf2 mutant (white) are the same as in Figure S9. (G) A wild-type seedling treated with 25 µM norflurazon and 450 µM lincomycin. (H) An enf2 seedling treated with 150 µM lincomycin. (I) A wild-type seedling treated with 450 µM lincomycin. Scale bars represent 1 mm (A–C, G–I) and 50 µm (D, E). (TIF) [file pgen.1003655.s010.tif]

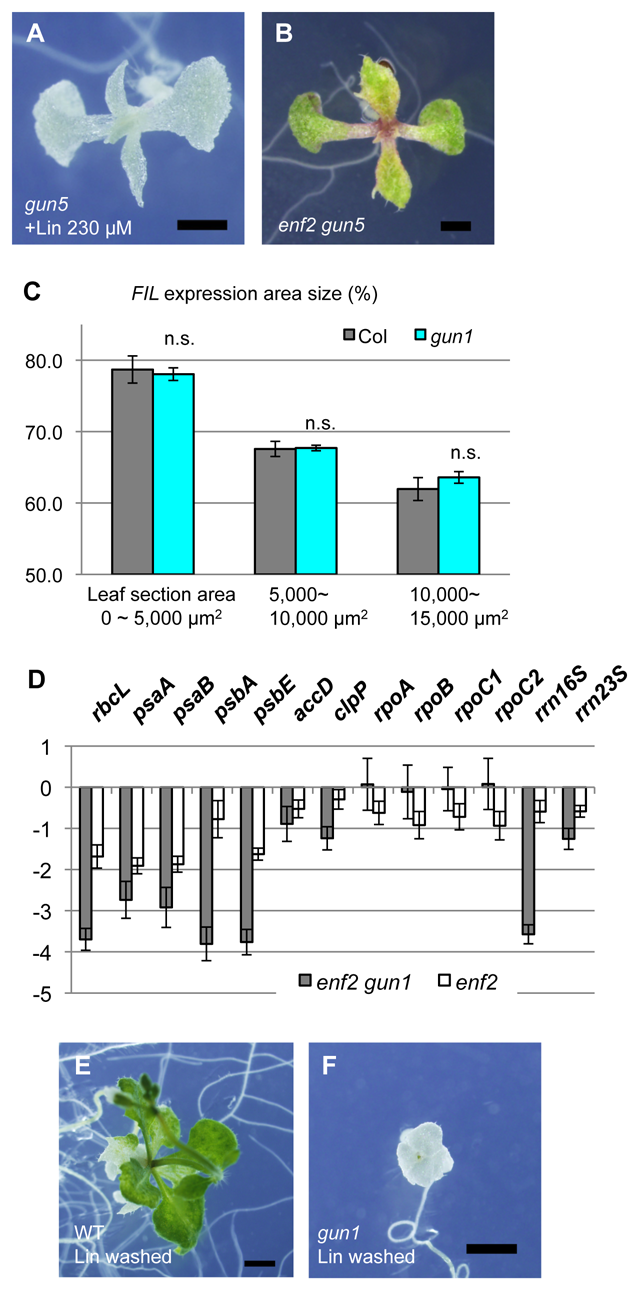

Supplement: Figure S11 — The involvement of plastid retrograde signal in the developmental effects from the plastid gene expression. (A) A gun5 seedling treated with 230 µM lincomycin (B) An enf2 gun5 seedling. (C) FIL-expression area sizes (%, y-axis) at different stages (grouped by section area sizes, x-axis) of the wild-type and gun1 leaf primordia. Bars indicate the standard errors. n.s., not significantly different (p≥0.05, t-test) between the wild type and gun1. (D) The qRT-PCR results showing the transcript abundance of plastid genes encoding proteins (left eleven) and 16S and 23S rRNA (right two) in enf2 gun1 (grey) shoot apex. The results were normalized to 18S rRNA, and the relative mutant values to the wild type are represented as log2 values. Error bars indicate standard errors. The data of enf2 mutant (white) are the same as in Figure S9. (E, F) The wild type and gun1 seedlings grown on the standard medium after growth on the lincomycin-containing medium for two weeks each. Scale bars represent 1 mm. (TIF) [file pgen.1003655.s011.tif]

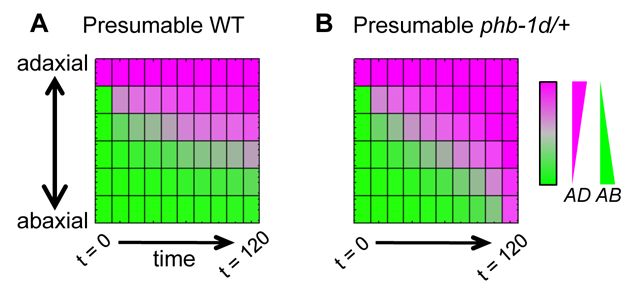

Supplement: Figure S12 — The model of mutual repression and mobility is applicable to simulation of phb-1d/+ phenotype. Computer simulation results with an assumed wild type parameter set (A): p1 = p2 = 0.1, r1 = 2.0, r2 = 1.8, d1 = d2 = 0.1, c1 = c2 = 2.0 and DAD = DAB = 0.1, and an assumed phb-1d/+ parameter set (B): p1 = p2 = 0.1, r1 = 2.0, r2 = 1.8, d1 = d2 = 0.1, c1 = 1.9, c2 = 2.0 and DAD = DAB = 0.1. Note that c1 value is smaller in B than in A. The adaxial-most cell was fixed in the AD-expressing state through each simulation, and the other abaxial five cells were set to be in the AB-expressing state as their initial conditions and followed the equations (1–4) during the simulations. (TIF) [file pgen.1003655.s012.tif]
