# Supplementary material for: Pattern Dynamics in Adaxial-Abaxial Specific Gene Expression Are Modulated by a Plastid Retrograde Signal during Arabidopsis thaliana Leaf Development
Source: PLoS Genet. 2013 Jul 25;9(7):e1003655. doi: 10.1371/journal.pgen.1003655 (PMC3723520; doi:10.1371/journal.pgen.1003655)
Supplement: Text S1 — The efficiency and spatio-temporal specificity of the CRE/loxP recombination. (PDF) [file pgen.1003655.s014.pdf]

Text S1

The efficiency and spatio-temporal specificity of the CRE/loxP recombination

When the plants harboring the *FILpro:CRE-GR 35Spro:loxP-Ter-loxP-VENUS* system were constantly treated with DEX from germination, the VENUS signal was detected in the whole or almost the whole leaves (Figure S3C, S3D) and in the abaxial half of flower primordia (Figure S3A). However, the signal was absent from the adaxial half of flower primordia and the apical meristem (Figure S3A). This result indicates that these VENUS-negative cells had not expressed *FIL* since germination, which is consistent with previous studies that showed the absence of *FIL* expression from apical meristem and adaxial half of flower primordia [1-2] (Figure S3B). From this result, we concluded that the CRE-loxP recombination specifically occurs with high efficiency in the *FIL*-expressing cells, but does not in the non-expressing cells.

The *FILpro:CRE-GR 35Spro:loxP-Ter-loxP-VENUS* plants were also grown on the DEX-free medium from the seeds to check the frequency of DEX-independent recombination. As a result, dotted patterns of VENUS-expressing cells were observed (Figure S3E, S3F), indicating that the DEX-dependent association between the CRE-GR and loxP site on the DNA is leaky in our system. Nonetheless, average sizes of these DEX-independent clones were only 0.77 percent of adaxial epidermis and 7.07 percent of abaxial epidermis in mature leaves (Figure S3E, S3F). Such frequencies are low enough to analyze the dynamic changes in the *FIL* expression pattern during the leaf development.

We also observed the VENUS expression pattern soon after the DEX application to the *FILpro:CRE-GR 35Spro:loxP-Ter-loxP-VENUS* plants grown on the DEX-free medium. The VENUS fluorescence showed dotted patterns at six hours after the DEX application (Figure S3G), and the patterns similar to that of *FILpro:GFP* at twelve hours after the application (Figure S3H). Therefore, the CRE/loxP recombination is efficiently induced in the *FIL*-expressing cells within half a day. Because the VENUS expression domain appeared to be slightly broader than that of *FILpro:GFP*, it was suggested that *FILpro:GFP* expression was repressed during the time from DEX application to the observation and/or that CRE has enough DNA recombinase activity at only low concentration at which GFP protein does not give detectable green fluorescence.

From these results, we concluded that our CRE/loxP system traces the cell lineage patterns of the *FIL*-expressing cells from the DEX application with enough efficiency and specificity.

References

1. Heisler MG, Ohno C, Das P, Sieber P, Reddy GV, et al. (2005) Patterns of auxin transport and gene expression during primordium development revealed by live imaging of the *Arabidopsis* inflorescence meristem. Curr Biol 15: 1899-1911.
2. Goldshmidt A, Alvarez JP, Bowman JL, Eshed Y (2008) Signals derived from *YABBY* gene activities in organ primordia regulate growth and partitioning of *Arabidopsis* shoot apical meristems. Plant Cell 20: 1217-1230.
